# Biotransformation of Cortisone with *Rhodococcus rhodnii*: Synthesis of New Steroids

**DOI:** 10.3390/molecules26051352

**Published:** 2021-03-03

**Authors:** Federico Zappaterra, Stefania Costa, Daniela Summa, Valerio Bertolasi, Bruno Semeraro, Paola Pedrini, Raissa Buzzi, Silvia Vertuani

**Affiliations:** 1 Department of Life Sciences and Biotechnology, University of Ferrara, Via L. Borsari 46, 44121 Ferrara, Italy; federico.zappaterra@unife.it (F.Z.); daniela.summa@unife.it (D.S.); pdp@unife.it (P.P.); raissa.buzzi@unife.it (R.B.); 2 Department of Chemical and Pharmaceutical Sciences, University of Ferrara, Via L. Borsari 46, 44121 Ferrara, Italy; valerio.bertolasi@unife.it; 3 GATE SRL, Via L. Borsari 46, 44121 Ferrara, Italy; bsemeraro@gategreen.it; 4 Department of Life Sciences and Biotechnology, Master Course in Cosmetic Science and Technology (COSMAST), University of Ferrara, Via L. Borsari 46, 44121 Ferrara, Italy; silvia.vertuani@unife.it

**Keywords:** biotransformation, cortisone, *Rhodococcus rhodnii*, steroids

## Abstract

Cortisone is a steroid widely used as an anti-inflammatory drug able to suppress the immune system, thus reducing inflammation and attendant pain and swelling at the site of an injury. Due to its numerous side effects, especially in prolonged and high-dose therapies, the development of the pharmaceutical industry is currently aimed at finding new compounds with similar activities but with minor or no side effects. Biotransformations are an important methodology towards more sustainable industrial processes, according to the principles of “green chemistry”. In this work, the biotransformation of cortisone with *Rhodococcus rhodnii* DSM 43960 to give two new steroids, i.e., 1,9β,17,21-tetrahydoxy-4-methyl-19-nor-9β-pregna-1,3,5(10)-trien-11,20-dione and 1,9β,17,20β,21-pentahydoxy-4-methyl-19-nor-9β-pregna-1,3,5(10)-trien-11-one, is reported. These new steroids have been fully characterized.

## 1. Introduction

Steroid drugs are used for various therapeutic applications and represent the most marketed drug category after antibiotics, with an annual production of over one million tons. To date, there are about 300 steroid-based drugs on the market, and this is constantly growing [1]. In this field, corticosteroids are a group of hormones produced by the adrenal gland cortex. This type of molecule is used for its anti-inflammatory, immunosuppressive properties and for its effects on metabolism. In particular, steroids belonging to the glucocorticoid class can control the metabolism of carbohydrates, lipids, and proteins, while mineralocorticoids control the concentration of electrolytes and, consequently, the amount of water present in the blood [2].

Glucocorticoids play an important role in anti-inflammatory and immunosuppressive therapy [3] and are widely used in the treatment of allergic reactions and inflammatory and autoimmune diseases, as well as in the prevention of rejection in transplants and the treatment of hematological neoplasia [4].

Unfortunately, the use of these drugs (also known as corticosteroids) has a long series of side effects. On the other hand, it is known that even small structural differences can significantly modify the power and duration of an action.

Besides, the glucocorticoids exhibit important interactions with drugs that affect the levels of potassium in the blood (e.g., diuretics and laxatives) [5] and with drugs that decrease cortisone in the blood (e.g., rifampicin, carbamazepine, phenobarbital, phenytoin, primidone, and lithium) [6,7]. The occurrence of such side effects is greater the higher the dosage and the duration of the treatment. Additionally, the side effects are more common in the case of systemic therapy (oral or injective) and rare for local therapies (with creams, ointments, and eye drops), where they are generally used at low dosages. Moreover, the glucocorticoids—in particular, cortisol and cortisone—are insoluble in water, and this limits their therapeutic use. Therefore, the steroidal compounds with anti-inflammatory action, which potentially do not present all the above-mentioned disadvantages, are investigated.

**Figure 1:**
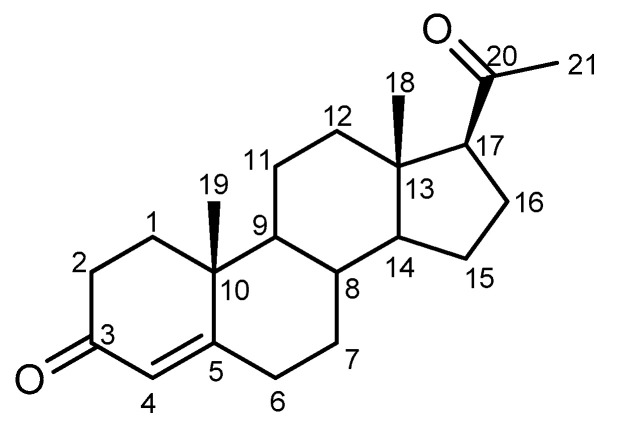
Chemical structure of progesterone.

In this field, to produce drugs with greater therapeutic activity and fewer side effects, the development of synthetic compounds deriving structurally from progesterone is of interest (Figure 1).

It is known that even minimal structural differences of these molecules can significantly change the potency and duration of corticoid actions. In this field, several patents describe steroid compounds, mainly characterized by the presence of a substituent group in position C_2_, with cellular modulation activity and antiproliferative and antiangiogenic effects [8]. These compounds are obtained through traditional chemical processes. In this context, to bypass the problems arising from chemical synthesis, the biotransformation reactions of steroid molecules have been studied in depth over the past 70 years [9]. This approach offers several advantages over chemical synthesis, such as the region- and/or stereospecificity of the rations and the eco-compatibility of the processes, as it works in mild reaction conditions and aqueous solvents. Therefore, the production of steroids and hormones is one of the best examples of the successful application of microbial technology in large industrial processes [10]. Research activities in this field began in the 1950s, following the identification of the pharmacological effects of cortisol and progesterone and with the identification of the enzyme hydroxylation activity in position C_11_ of the microbial genus *Rhizopus* [11]. Since then, multiple microbial bioconversion processes of steroids and sterols have been reported in the literature; these biotransformations have provided adequate tools for the large-scale production of analogs of natural or modified steroids [12,13].

In this field, the ability of certain microorganisms to produce various metabolites is well-known and not readily achievable by conventional methods, and this practice has proven to be indispensable for the pharmaceutical industry [14].

To maximize the yield of the reaction, understood as the total conversion of the substrate into the desired product, selected strains of a particular microorganism are generally transformed to optimize the expression of the genes of interest and/or block certain metabolic pathways to accumulate the pursued intermediates.

In this context, the ability of *Actinobacteria* to biotransform and, in some cases, to partially or totally degrade steroid rings or side chains has turned out to be of great interest [15,16].

*Actinobacteria* (also called *Actinomycetes* or *Mycobacteria*, as bacteria similar to filamentous fungi) are a group of Gram-positive bacteria (with the sole exception of *Actinoplanes*) that includes five subclasses and 35 families [17]. These strains have been widely studied and their biotransformation capacities confirmed in various types of transformations of steroid rings as the reduction of C-C and C-O double bonds, oxidation of alcoholic functions, dehydrogenation, hydroxylation, and in many cases, also in lateral-chain degradation processes or the total degradation of the steroid ring [18].

In particular, *Rhodococcus*, which belong to the order of Actinomycetales, are Gram-positive, forced aerobic, nonmobile, nonsporulating bacteria and are usually found in soil and aquatic environments and are very important for industrial use. Several studies have shown that these strains can grow both in mesophilic [19] and in psychrophilic conditions [20].

*Rhodococci* are also able to degrade various organic compounds, including ones highly toxic and recalcitrant; moreover, they are equipped with enzymes that catalyze biologically relevant reactions such as the biodesulfurization of fossil fuels [21], the degradation of polychlorinated biphenyls (PCBs) [22], and the use of a large variety of other organic compounds as sources of energy [19]. They are also able to degrade halogenated hydrocarbons with long or short chains [23] and to metabolize numerous halogenated differently substituted aromatic and heteroaromatic compounds [24], bioactive steroids [25], and acrylamide [26]. *Rhodococci* are also known for their ability to biotransform a wide range of steroids via hydroxylation or Bayer-Villiger oxidation, while the *Corynebacterium* and *Nocardia* genera are known to provide dehydrogenation products [18].

In previous works, the biotransformations of bile acids with *Rhodococcus ruber* to give new 9,10-secosteroids [15], and of cortisone and cortisol with *Rhodococcus coprophilus* to give Δ^1^-dehydrogenation [27] products, have been reported. These works derive from a preliminary screening of different strains of *Rhodococcus*, from which it emerged that *Rhodococcus rhodnii* was able to biotransform cortisone (**1**) by producing compounds different from the classic biotransformation products (i.e., prednisone or hydroxyl reduction reactions in position C_20_) [27].

**Scheme 1:**
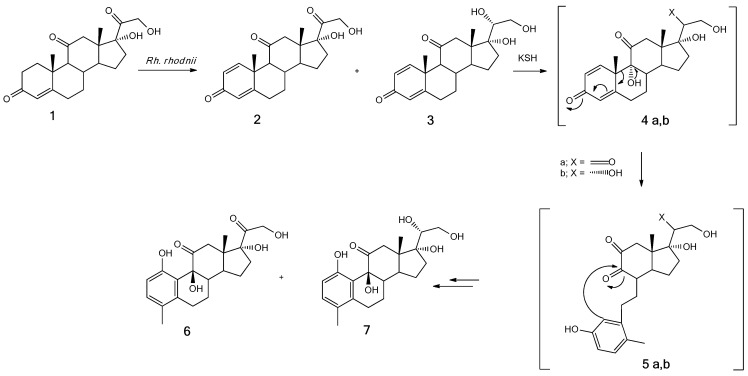
Biotransformation of cortisone (**1**) with *Rhodococcus rhodnii*.

In this work, the biotransformation of cortisone (**1**) with *Rhodococcus rhodnii* to give new steroid compounds, i.e., 1,9β,17,21-tetrahydroxy-4-methyl-19-nor-9β-pregna-1,3,5(10)-trien-11,20-dione (**6**) and 1,9β,17,20β,21-pentahydroxy-4-methyl-19-nor-9β-pregna-1,3,5(10)-trien-11-one (**7**), is reported (Scheme 1).

## 2. Results

### 2.1. Biotransformation of Cortisone with Rhodococcus rhodnii (24 h)

Semipreparative cultures were necessary to isolate and identify the biotransformation products. Although the biotransformation time (24 h) was rather short, the yields of the product were almost quantitative; in fact, no residual cortisone was observed, and 1,9β,17,21-tetrahydoxy-4-methyl-19-nor-9β-pregna-1,3,5(10)-trien-11,20-dione **6** (0.146 ± 0.07 g, 70%) and 1,9β,17,20β,21-pentahydoxy-4-methyl-19-nor-9β-pregna-1,3,5(10)-trien-11-one) **7** (0.041 ± 0.004 g, 20%) were obtained. In this case, the product recovery yield was not quantitative, as the downstream processes of extraction and purification of the products on a silica gel column led to a loss of about 5-10%. An improvement in yield could be obtained by trying to optimize the recovery process of the biotransformation products.

As regards the course of the reaction, samples were taken at 0, 2, 4, 6, 8, and 24 h from the administration of the substrate (**1**). After 2 h from the TLC (Thin Layer Chromatography) analysis, only the appearance of a band corresponding to prednisone (**2**) was shown, while the cortisone band was still very evident. At 4 h, a further band appeared in correspondence with the 20β-hydroxy prednisone (**3**) standard. After 6 h from the administration of the substrate, the bands of products **2** and **3** were clearly visible, even if a large part of the unreacted cortisone remained, a situation which remained almost unchanged even at 8 h. After 24 h of biotransformation, the only visible bands were those relating to products 1,9β,17,21-tetrahydroxy-4-methyl-19-nor-9β-pregna-1,3,5(10)-trien-11,20-dione (**6**) and 1,9β,17,20β,21-pentahydroxy-4-methyl-19-nor-9β-pregna-1,3,5(10)-trien-11-one (**7**).

**Figure 2:**
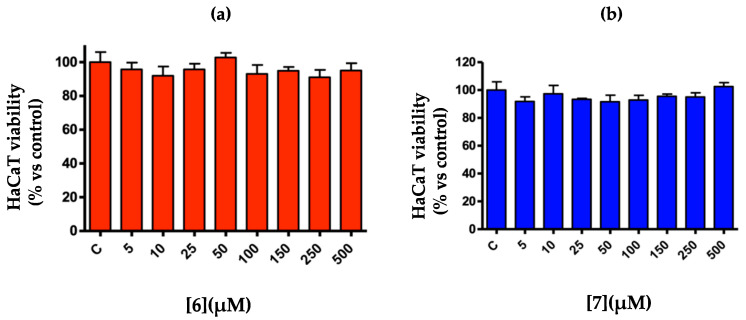
3-(4,5-Dimethylthiazol-2-yl)-2,5-diphenyltetrazolium bromide (MTT) test about the cell viability of the aneuploid immortal keratinocyte c ell line after exposure to product **6** (**a**) and product **7** (**b**). The values were expressed as a percentage with respect to the control (C = 100%) to the vehicle alone and reported as mean ± SEM.

The structure of compound **6** was determined by NMR analysis (Table 1 and Table 2), mass spectrometry, IR, and X-ray investigation (Table 3 and Figure 2). The only difference between compounds **6** and **7** was the reduction of the C_20_-carbonyl group.

The stereochemistry of the C_20_-hydroxyl group was determined by the comparison of the commercial standard spectrum of 17α,20β,21-trihydroxy-1,4-pregnadiene-3,11-dione (20β-hydroxy-prednisone) **3** that was isolated as an intermediate [28]. Based on the degradation pathway of cortisone, we hypothesized the formation mechanism of products **6** and **7**, as shown in Scheme 1.

Compounds **6** and **7** showed substantial structural variations concerning cortisone—in particular, the aromatization of the A ring and the displacement of the angular methyl group from the C_19_ position (δ 1.32 ppm) in cortisone to the C_4_ position in the new compounds (δ 2.08 and 2.09 ppm for **6** and **7**, respectively). The aromatization of ring A was shown by the presence of the signals C_2_-H (δ 6.90 ppm) and C_3_-H (δ 6.40 ppm) for product **6** and by the absence of signals C_1_-H and C_9_-H present in cortisone. Minimal differences were present in compound **7** (C_2_-H δ 6.45 ppm and C_20_-H δ 3.48 ppm). A further diagnostic signal of the change in the structure of ring A was represented by the signal shift relative to the hydrogen atom in the C_4_ position of the cortisone, as this atom was replaced by the methyl group (C_19_) following the rearrangement of the molecule after hydroxylation and the opening of ring B.

As far as the hydroxyl groups visible from the NMR spectra in DMSO–*d*_6_ are concerned, the appearance of the signal relating to the OH group in position C_20_ in product **7** at 4.20 ppm was noted, the signal absent in compounds **1** and **6** as they presented a group in this position carbonyl. Further confirmation of this data was provided by the signal at 3.48 ppm of the ^1^H-NMR spectrum of product **7**, absent in the other two spectra (see Supplementary Materials).

As prednisone has proven to be four to five times more active than cortisone and hydrocortisone [29] due to the alteration of the conformation of ring A, compounds **6** and **7** could also have an anti-inflammatory capacity comparable or superior to the starting substrate; this would be a huge advantage in terms of the amount of drugs that should be given. Furthermore, products **6** and **7**, inside the steroid structure, present ring A in a phenolic form; this structure could also confer antioxidant properties to these molecules.

Based on the suggested biotransformation pathway of cortisone, products **2** and **3** are potential precursors of products **6** and **7**, as shown in Scheme 1.

In this mechanism, the Δ^1^-dehydrogenation of cortisone (catalyzed by a Δ^1^-hydroxysteroid dehydrogenase) and the reduction of the hydroxyl group in the C_20_ position, catalyzed by a 20β-hydroxysteroid dehydrogenase, were the first reactions involved to give prednisone **2** and 20β-hydroxy-prednisone **3**. Subsequently, the hydroxylation of the C_9_ position by the 3-ketosteroid-9α-hydroxylase (KSH) afforded the intermediates **4a**, **b** that, by the spontaneous retro-aldol cleavage of ring B, gave the aromatic 9,10-secosteroids **5a**, **b**. These intermediates were not isolated. In particular, we highlighted the nucleophilic attack of the benzenic ring on the carbonyl in the C_9_ position with the formation of the ring B and the recovery of the hydroxyl function in C_9_, as shown by the signals at 4.74 and 4.68 ppm for products **6** and **7**, respectively.

### 2.2. Biotransformation of Cortisone with Rhodococcus rhodnii (6 h)

The objective of carrying out biotransformations at short times was to confirm the first biotransformation step of compounds **2** and **3** and the stereochemistry of the hydroxyl group present in position C_20_ of the new product **7**; for this reason, the reaction was stopped after only 6 h.

Compound **3** is known in the literature and is commercially available. As was done in a previous study, the purification of intermediate **3** corresponded to 20β-prednisone. The stereochemistry of the C_20_ reduction and, consequently, of product **3**, was confirmed by comparing the ^1^H-NMR data (i.e., 4.61 (C_20_-H) and 4.52 (C_21_-H) ppm in C_5_D_5_N) and ^13^C-NMR data (i.e., 65.0 (C_21_) and 72.5 (C_20_) ppm) with the data recorded for the commercially available compound.

This product could be an interesting derivative of the bioactive prednisone. The yields obtained in these biotransformations, although of relative importance concerning the purpose of this experiment, were as follows: prednisone 2 (0.06 ± 0.003 g, 30%), 20β-hydroxy-prednisone 3 (0.02 ± 0.002 g, 10%), and unreacted cortisone 1 (0.12 ± 0.005 g, 60%).

As can be seen from the yields, given the short times of biotransformation, 60% of cortisone was not yet biotransformed.

### 2.3. 3-(4,5-Dimethylthiazol-2-yl)-2,5-Diphenyltetrazolium Bromide (MTT) Assays

Product **6** and product **7** were tested for the determination of cell viability on the human HaCaT keratinocyte lines. The test was conducted by exposing HaCaT cells to different concentrations (from 5 to 500 µM) of the “product **6** and **7**” test samples for 24 h at 37 °C, and then, cell viability was measured by means of the MTT test. The negative control (0.1% DMSO culture medium) was tested simultaneously with the substances. Both substances examined were not cytotoxic at any of the tested concentrations, since they did not decrease the vitality of the keratinocytes compared to the control culture.

## 3. Discussion

To bypass all or part of the side effects deriving from the cortisone therapies, the pharmaceutical industry is increasingly looking for new compounds with therapeutic characteristics similar to those of cortisone but with fewer side effects.

Due to the numerous harmful and dangerous effects of the reagents used in the chemical synthesis of new compounds, research is increasingly turning towards greener approaches. To overcome all the problems deriving from chemical synthesis, the potential of microbial steroid biotransformation has been known for several decades, as its application offers several advantages over chemical synthesis. 

In the present work, the bacterium *R. rhodnii* biotransformed cortisone **1** in 24 h to produce the new steroids **6** and **7**.

**Scheme 2:**
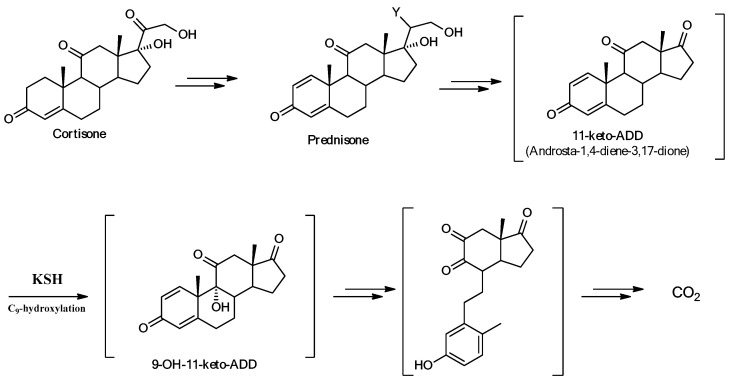
Degradation pathway of cortisone by *Actinobacteria* [18].

The proposed mechanism of formation of products **6** and **7** (Scheme 1) in the initial steps was supported and followed partially the degradation pathway of cortisone by *Actinobacteria* [18] (Scheme 2).

Steroids with a 3-oxo-4-ene structure, such as cortisone, are subjected to the cleavage of ring B. The Δ^1^-dehydrogenation of cortisone to give prednisone is the initial stage that leads to the complete elimination of the C_17_ chain with the 11-keto-ADD (androstadienedione) formation. This is a classic step in the degradation of other steroids, such as sitosterol, testosterone, and bile acids [30]. 11-Keto-ADD is subjected to hydroxylation at the C_9_ position catalyzed by 3-ketosteroid-9α-hydroxylase (KSH), a two-component iron–sulfur-containing monooxygenase [31,32,33]. This reaction leads to the spontaneous cleavage of the ring B by a retro-aldol cleavage and the formation of the aromatic 9,10-secosteroids, which undergo further degradation [34].

This degradation pathway sustained the proposed mechanism of C_9_-hydroxylation of prednisone **2** and prednisolone **3**, whose presence was confirmed stopped by the biotransformation of cortisone after 6 h. The C_9_-hydroxylated intermediates **4a**, **b** spontaneously rearranged to secosteroids **5a**, **b.** The nucleophilic attack of the aromatic ring on the carbonyl in C_9_ ld to the subsequent B ring closure with the formation of new compounds **6** (70%) and **7** (20%). The yields obtained did not correspond to 100%, as it was classic that, in the extraction and purification of the products, there was a weight loss of about 5–10%. Furthermore, the path that led to the degradation of the compounds probably began at this level; in fact, some studies have been conducted in which the duration of the biotransformation was extended up to 72 h, with a decrease in the yield of the products obtained, so we hypothesized that the degradation mechanism started after about 24 h [18].

The structures of compounds **6** and **7** allowed us to think that they could maintain an anti-inflammatory capacity, as they had a carbonyl function in position C_11_. Furthermore, products **6** and **7**, inside the steroid structure, presented ring A in a phenolic form; this structure could also confer antioxidant properties to these molecules.

To date, the biotransformation approach is the only synthesis method of these molecules; therefore, if in the future, it will be demonstrated that these new steroids have therapeutic activity, it will be possible to produce them using a method that follows the guidelines of “green chemistry”.

About the reduction product at the level of the carbonyl group in C_20_, the stereochemistry of the hydroxyl group was carried out by comparing the NMR spectra of its precursor with a commercial standard.

## 4. Materials and Methods

### 4.1. Chemicals and Rhodococcus Strain

Cortisone was purchased from Sigma-Aldrich (St. Louis, MO, USA). Plate count broth (PCB) is commercially available (Oxoid, (Basingstoke, UK)). 20β-hydroxy-prednisone was purchased from Toronto Research Chemical Inc. (Toronto, ON, Canada).

*Rhodococcus rhodnii* (DSM 43960) was purchased from the DSMZ (Leibniz Institute DSMZ—German Collection of Microorganisms and Cell Cultures GmbH, Braunschweig Germany) company. The master cell bank of every strain was maintained at −20 °C in cryovials in plate count broth medium (1 mL) mixed with glycerol (0.5 mL) as a cryoprotectant agent. The working cell bank was conserved at 4 °C in plate count agar (PCA) slants for 6 months and used for seed cultures.

### 4.2. Analytical Methods

Melting points were uncorrected and were determined on a 510 Buchi melting point instrument. The ^1^H, ^13^C, and DEPT NMR spectra were recorded with a Varian Gemini 300 operating at 300 MHz (^1^H) and 75 MHz (^13^C) or with a Varian Mercury Plus 400 operating at 400 MHz (^1^H) and 100 MHz (^13^C), respectively. The chemical shifts were referenced to the residual solvent signals (CDCl_3_: δ_H_ 7.26, δ_C_ 77.0; CD_3_OD: δ_H_ 3.34, δ_C_ 49.0; and DMSO-*d*6: δ_H_ 2.49, δ_C_ 39.9). The 2D NMR experiments (COSY, HMQC, and HMBC) were obtained with a Varian Mercury Plus 400, operating at 400 MHz (^1^H) and 100 MHz (^13^C), and were processed using the manufacturer’s software. Infrared spectra were acquired with a Perkin-Elmer Spectrum 100 FTIR. Mass spectra were recorded using an LCQ Duo (TermoQuest, San Jose, CA, USA), equipped with an electrospray ionization (ESI) source. Optical rotations were measured at 20 °C in the stated solvent using a Perkin-Elmer model 241 polarimeter. TLC were performed on precoated silica gel plates (thickness 0.25 mm, Merck), and the phosphomolybdic acid solution was used as the spray reagent to visualize the steroid spots. Silica gel (Fluka, Kiesegel 60, 70–230 mesh) was used for preparative column chromatography.

### 4.3. Biotransformation of Cortisone **1** with Rhodococcus rhodnii (24 h)

A loopful of *Rhodococcus rhodnii* from a culture on plate count agar (PCA) containing glucose (1 g/L), yeast extract (2.5 g/L), tryptone (5 g/L), and agar (15 g/L) was inoculated in three 50-mL Erlenmeyer flasks, closed with cotton plugs, in sterile plate count broth (PCB) (20 mL) containing glucose (1 g/L), yeast extract (2.5 g/L), and tryptone (5 g/L) and incubated at 30 °C and 110 rpm in an orbital shaker for 48 h. The whole cultures were introduced into 500-mL Erlenmeyer flasks containing 200 mL of sterile PCB, and after 48 h incubation at 30 °C and 110 rpm, cortisone (0.2 g) in DMSO (2 mL) was added, and the culture was maintained in the same conditions for 24 h. All tests were carried out in triplicate for statistical significance. A blank sample was also incubated containing all the reagents but no bacteria. The reaction was monitored by withdrawing samples (1 mL) every 6 h up to 24 h of reaction and followed by TLC analysis (ethyl acetate as the eluent). After 24 h, the cells were removed by centrifugation (5242 RCF, 20 min), and the supernatant was extracted with ethyl acetate (3 × 200 mL). The organic layer was dried over anhydrous Na_2_SO_4_, the solvent evaporated, and the crude mixture purified on a chromatographic column (silica gel; ethyl acetate as the eluent). The biotransformation yields were obtained by working out the percentage of the ratio between the moles of the product obtained compared to the moles of the administered substrate.

### 4.4. Biotransformation (6 h) of Cortisone **1** with Rhodococcus rhodnii

The biotransformations of cortisone **1** with *Rhodococcus rhodnii* at 6 h were carried out following the same protocols used for those at 24 h with the differences that the TLC analyses were performed using ethyl acetate/cyclohexane 50/50 as the eluent, and the crude mixture was purified on a chromatographic column (silica gel; ethyl acetate/cyclohexane 50/50 as the eluent). 

### 4.5. Crystal Structure Determination

The crystal data of compound **6** were collected at room temperature using a Nonius Kappa CCD diffractometer with graphite monochromated Mo-Kα radiation. The datasets were integrated with the Denzo-SMN package [35] and corrected for Lorentz and polarization effects. The structure was solved by direct methods using the SIR97 [36] system of the programs and refined using full-matrix least-squares with all nonhydrogen atoms anisotropically and hydrogens included on calculated positions, riding on their carrier atoms, except those linked to the oxygen atoms that were refined isotropically. Compound **6** crystallized in the monoclinic non-centrosymmetric space group, *P2*_1_, and the absolute configuration was determined reliably from the crystallographic data because of the calculated Flack parameter of 0.1 [37].

**Figure 3:**
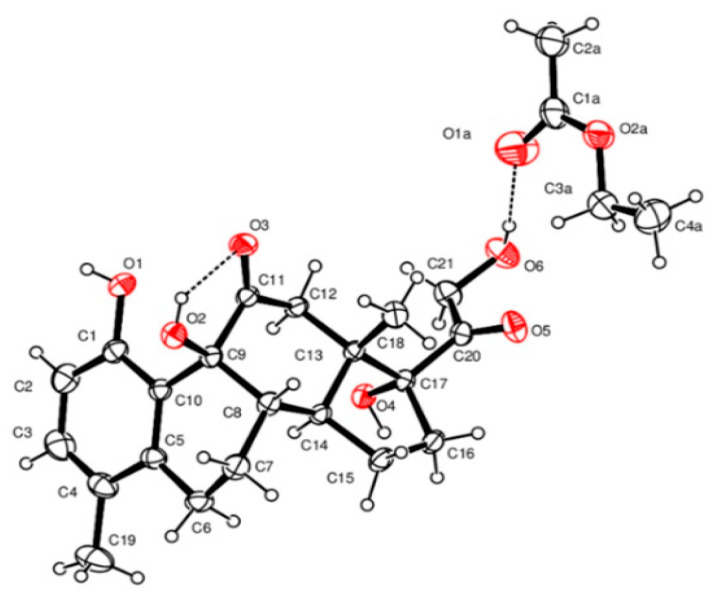
An ORTEP view of compound **6** showing the thermal ellipsoids at a 30% probability level. The crystallographic asymmetric unit is built up by a molecule of compound **6** and a molecule of solvent ethyl acetate.

All calculations were performed using SHELXL-2018/3 [37] and PARST [38] implemented in the WINGX system of the programs. The crystal data are given in Table 1. The ORTEP (Oak Ridge Thermal-Ellipsoid Plot Program) [39] view of the structure is shown in Figure 3.

### 4.6. Compound Data

17α,21-dihydroxy-1,4-pregnadiene-3,11,20-trione (prednisone) **2** showed the following: m.p. (melting point) 234 °C; ^1^H-NMR (CDCl_3_): see Table 2; and ^13^C-NMR (CDCl3): see Table 3. 

17α,20β,21-trihydroxy-1,4-pregnadiene-3,11-dione (20β-hydroxy-prednisone) **3** showed the following: m.p. 240–242 °C; ^1^H-NMR (CDCl_3_): see Table 2; and ^13^C-NMR (CDCl3): see Table 3. 

1,9β,17,21-tetrahydroxy-4methyl-19-nor-9β-pregna-1,3,5(10)-trien-11,20-dione (**6**) (Scheme 1) showed the following: m.p. 91–92 °C; [α]D: + 64,6 (c 1.0, Methanol); IR νmax (KBr) 3338, 2939, 1708, 1590; ESI-MS (negative ion mode): calcd for C_21_ H_26_ O_6_: 374.17. Found, *m*/*z* (% relative to the base peak): 373.27 (100) [M − H+]; ^1^H-NMR (DMSO-*d*6): see Table 4; and ^13^C-NMR (DMSO-*d*6): see Table 5.

1,9β,17,20β,21-pentahydroxy-4-methyl-19-nor-9β-pregna-1,3,5(10)-trien-11-one (**7**) (Scheme 1) showed the following: m.p. 141–142 °C; [α]D: +23.3 (c 1.0, Methanol); IR νmax (KBr) 3358, 2931, 1698, 1590; ESI-MS (negative ion mode): calcd for C_21_ H_28_ O_6_: 376.18. Found, *m*/*z* (% relative to the base peak): 375.13 (100) [M–H+]; ^1^H-NMR (DMSO-*d*6): see Table 4; and ^13^C-NMR (DMSO-d_6_): see Table 5.

### 4.7. Cytotoxic Activity

HaCaT cells were cultured using Ham’s F-12, fetal bovine serum, DMEM high glucose, penicillin/streptomycin, and L-glutamine. Cells were incubated at 37 °C for 24 h in 95% air/5% CO_2_ until 80% confluency. 3-(4,5-dimethylthiazol-2-yl)-2,5-diphenyltetrazolium bromide (MTT) colorimetric assay was performed to estimate cell viability [40]. Cells were seeded in 96-well plates at a density of 2 × 104 cells/well in 200-μL complete medium for 24 h. Different concentrations (from 5 to 500 μm) of products **6** and **7** were used as cell treatments. After 72 h, the medium was removed and washed with PBS twice; 20 μL of MTT (5 mg/mL) was then added in each well, and the plates were incubated for 4 h at 37°C. The medium was removed and replaced with 100-μL DMSO to dissolve the formazan crystals. The extent of MTT reduction was measured spectrophotometrically at 570 nm using a microplate reader (680 XR, Bio-Rad, Hercules, CA, USA).

## 5. Conclusions

This work focused on the synthesis of two new compounds resulting from the biotransformation of cortisone catalyzed by *Rhodococcus rhodnii* (DSMZ 43960): 1,9β,17,21-tetrahydroxy-4-methyl-19-nor-9β-pregna-1,3,5(10)-trien-11,20-dione (**6**) and 1,9β,17,20β,21-pentahydroxy-4-methyl-19-nor-9β-pregna-1,3,5(10)-trien-11-one (**7**).

The time of biotransformation (24 h) was rather short, and the yield of the product was almost quantitative, as no residual cortisone was observed.

To confirm the correct chemical structure of these new products, X-ray studies were carried out on product **6**, while for the determination of the stereochemistry of the hydroxyl group in the C_20_ position of product **7**, a comparison of the NMR spectra of its precursor with the reference commercial standard was carried out.

In this work, a green synthesis method of these new compounds was proposed, and currently, it is the only strategy that can be used both from the biotransformation point of view and from that of the chemical synthesis.

The chemical structures of these new products present peculiarities that suggest potential biological and therapeutic activity. The preliminary cytotoxicity tests obtained by MTT assay gave promising results for the use of these new derivatives, even at the highest concentrations tested. Further studies will be focused on the evaluation of the anti-inflammatory activity and the potential antioxidant activity.

## 6. Patents

WO2017037081A1 is the patent resulting from the work reported in this manuscript.

## Figures and Tables

**Table 1:** Crystallographic data.

Crystallographic Data	Compound 6
Formula	C_21_H_26_O_6_, C_4_H_8_O_2_
M	462.52
Space group	*P2*_1_
Crystal system	Monoclinic
a/Å	7.9730(2)
b/Å	16.3719(3)
c/Å	9.4380(3)
α/°	90.00
β/°	100.9145(8)
γ/°	90.00
U/Å^3^	1209.69(5)
*Z*	2
T/K	295
D_c_/g cm^−3^	1.270
F(000)	496
μ(Mo-Kα)/mm^−1^	0.094
Unique Reflections	6576
R_int_	0.0366
Obs. Refl.ns [I ≥ 2σ(I)]	5759
θ_min_–θ_max_/°	3.71−30.00
hkl ranges	−11,11;−21,22;−13,13
R(F^2^) (Obs. Refl.ns)	0.0426
wR(F^2^) (All Refl.ns)	0.1143
No. Variables	318
Goodness of fit	1.043
Δρ_max_; Δρ_min_ /e Å^−3^	0.232; −0.022
CCDC Deposition N.	1909504

**Table 2:** ^1^H-NMR data of **2** and **3** at 400 MHz in CDCl_3_, δ in ppm, and J in Hz.

	2	3
H-C(1)	7.63 (d, *J* = 10.2 Hz)	7.67 (d, 1H, *J* = 10.2 Hz)
H-C(2)	6.19 (dd, *J* = 10.2 and 1.8 Hz)	6.18 (dd, *J* = 10.3 and 1.9 Hz)
H-C(3)	--	--
H-C(4)	6.07 (t, *J* = 1.8 Hz)	6.06 (t, *J* = 1.26 Hz)
H-C(5)	--	--
H-C(6)	2.01 (m)	1.92 (m)
H-C(7)	1.73 (m)	1.81 (m)
H-C(8)	1.98 (m)	1.93 (m)
H-C(9)	1.99 (d, *J* = 3.1 Hz)	2.45 (m)
H-C(10)	--	--
H-C(11)	--	--
H-C(12)	2.38 (m)	2.52 (m)
H-C(13)	--	--
H-C(14)	2.06 (m)	1.90 (m)
H-C(15)	1.94 (m)	1.82 (m)
H-C(16)	1.92 (m)	2.01 (m)
H-C(17)	1.69 (m)	1.60 (m)
H-C(18)	0.67 (s)	0.81 (s)
H-C(19)	1.42 (s)	1.43 (s)
H-C(20)	--	3.76 (br s) (4.61, m in C_5_D_5_N)
H-C(21)	4.24 (d, *J* = 20 Hz)	3.76 (br s) (4.52, m in C_5_D_5_N)

**Table 3:** ^13^C-NMR data of compounds **2** and **3** at 400 MHz in CDCl_3_, δ in ppm.

	2	3
H-C(1)	158.2	158.4
H-C(2)	127.8	127.7
H-C(3)	188.6	188.7
H-C(4)	124.7	124.6
H-C(5)	170.9	171.2
H-C(6)	34.9	35.0
H-C(7)	33.3	34.8
H-C(8)	37.4	37.7
H-C(9)	61.1	61.3
H-C(10)	44.1	44.1
H-C(11)	211.4	213.3
H-C(12)	51.1	52.7
H-C(13)	52.3	52.8
H-C(14)	50.8	49.5
H-C(15)	24.1	24.5
H-C(16)	35.2	33.4
H-C(17)	89.1	85.0
H-C(18)	16.2	15.9
H-C(19)	19.3	19.3
H-C(20)	212.7	72.5
H-C(21)	67.8	65.0

**Table 4:** ^1^H-NMR data of compounds **1**, **6**, and **7** at 400 MHz in DMSO-*d*6, δ in ppm, and *J* in Hz.

	**1**	**6**	**7**
H-C(1)	2.54 (m)/1.63 (m)	--	--
H-C(2)	1.91	6.90 (d, *J* = 8.1)	6.90 (d, *J* = 8.1)
H-C(3)	--	6.40 (d, *J* = 8.1)	6.45 (d, *J* = 8.1)
H-C(4)	5.61 (s)	--	--
H-C(5)	--	--	--
H-C(6)	2.26 (m)	2.45 (m) ^c^/2.52 (m) ^c^	2.62 (m) ^e^/2.51 (m) ^e^
H-C(7)	1.91 (m)	2.12 (m)/1.65 (m) ^d^	2.15 (m)/1.60 (m) ^f^
H-C(8)	1.91 (m)	1.62 (m) ^d^	1.58 (m) ^f^
H-C(9)	2.12 (m)	--	--
H-C(10)	--	--	--
H-C(11)	--	--	--
H-C(12)	2.85 (d, J = 10.8) ^a^/1.94 (d, J = 10.8) ^b^	2.81 (d, J = 10.6) ^a^/2.03 (d, J = 10.6) ^b^	2.68 (d, J = 10.6) ^a^/2.40 (d, J = 10.6) ^b^
H-C(13)	--	--	--
H-C(14)	2.33 (m)	2.50 (m) ^c^	2.25 (m)
H-C(15)	1.76 (m)/1.32 (m)	1.69 (m) ^a,d^/1.24 (m) ^b^	1.60 (m)^b,f^/1.15 (m) ^a^
H-C(16)	2.64 (m)/1.63 (m)	2.58 (m) ^b,c^/1.50 (m) ^a^	1.75 (m)^b^/1.45 (m) ^a^
H-C(17)	--	--	--
H-C(18)	0.45 (s)	0.44 (s)	0.65 (s)
H-C(19)	1.32 (s)	2.08 (s)	2.09 (s)
H-C(20)	--	--	3.48 (br s)
H-C(21)	4.44 (dd, J = 19.3, 6.6)/4.08 (dd, J = 19.3, 5.3)	4.36 (dd, *J* = 19.2, 6.4)/3.98 (dd, *J* = 19.2, 5.3)	3.35 (m) ^g^/3.30 (m) ^g^
H-O(3)	--	9.22 (s)	9.15 (s)
H-O(9)	--	4.74 (s)	4.68 (s)
H-O(17)	5.57 (s)	5.42 (s)	3.85 (s)
H-O(20)	--	--	4.20 (br d, *J* = 6.5)
H-O(21)	4.65 (dd, J = 6.3, 5.1)	4.66 (dd, *J* = 6.4, 5.3)	4.38 (br s)

^a^ α proton; ^b^ β proton; and ^c, d, e, f, and g^ overlapping multiplets.

**Table 5:** ^13^C-NMR data of compounds **1**, **6**, and **7** at 100 MHz in DMSO-*d*6, δ in ppm.

	**1**	**6**	**7**
C(1)	31.3	129.4	129.7
C(2)	33.8	112.2	112.7
C(3)	198.7	152.9	153.6
C(4)	124.1	123.5	124.3
C(5)	169.6	135.8	136.4
C(6)	32.4	22.1	22.6
C(7)	32.0	19.6	20.3
C(8)	36.4	41.9	42.9
C(9)	61.49	75.6	76.0
C(10)	38.2	126.0	126.3
C(11)	210.7	212.0	214.2
C(12)	50.5	44.3	46.4
C(13)	50.8	50.9	52.3
C(14)	49.5	42.2	41.2
C(15)	23.2	22.1	23.1
C(16)	34.0	33.5	34.5
C(17)	88.1	87.1	83.4
C(18)	15.9	14.4	14.7
C(19)	17.3	18.7	19.2
C(20)	211.9	211.0	75.4
C(21)	66.6	65.6	63.8

## Data Availability

The data presented are available in the manuscript Supplementary Materials.

## Supplementary Materials

The following are available online: Figure S1: ^1^H-NMR product 6. Figure S2: ^13^C-NMR product 6. Figure S3: DEPT-NMR product 6. Figure S4: COSY-NMR product 6. Figure S5: Focused COSY-NMR product 6. Figure S6: HMBC-NMR product 6. Figure S7: HMQC-NMR product 6. Figure S8: MS spectroscopy of product 6. Figure S9: IR spectroscopy of product 6. Figure S10: ^1^H-NMR product 7. Figure S11: ^13^C-NMR product 7. Figure S12: Focused ^13^C-NMR product 7. Figure S13: DEPT-NMR product 7. Figure S14: COSY-NMR product 7. Figure S15: HMBC-NMR product 7. Figure S16: focused HMBC-NMR product 7. Figure S17: HMQC-NMR product 7. Figure S18: MS spectroscopy of product 7. Figure S19: IR spectroscopy of product 7.

## Sample Availability

Samples of compounds **6** and **7** are available from the authors.
