# Supplementary material for: Biotransformation of Cortisone with Rhodococcus rhodnii: Synthesis of New Steroids
Source: Molecules. 2021 Mar 3;26(5):1352. doi: 10.3390/molecules26051352 (PMC7962003; doi:10.3390/molecules26051352)
Supplement: Supplementary file 1 [file molecules-26-01352-s001.pdf]

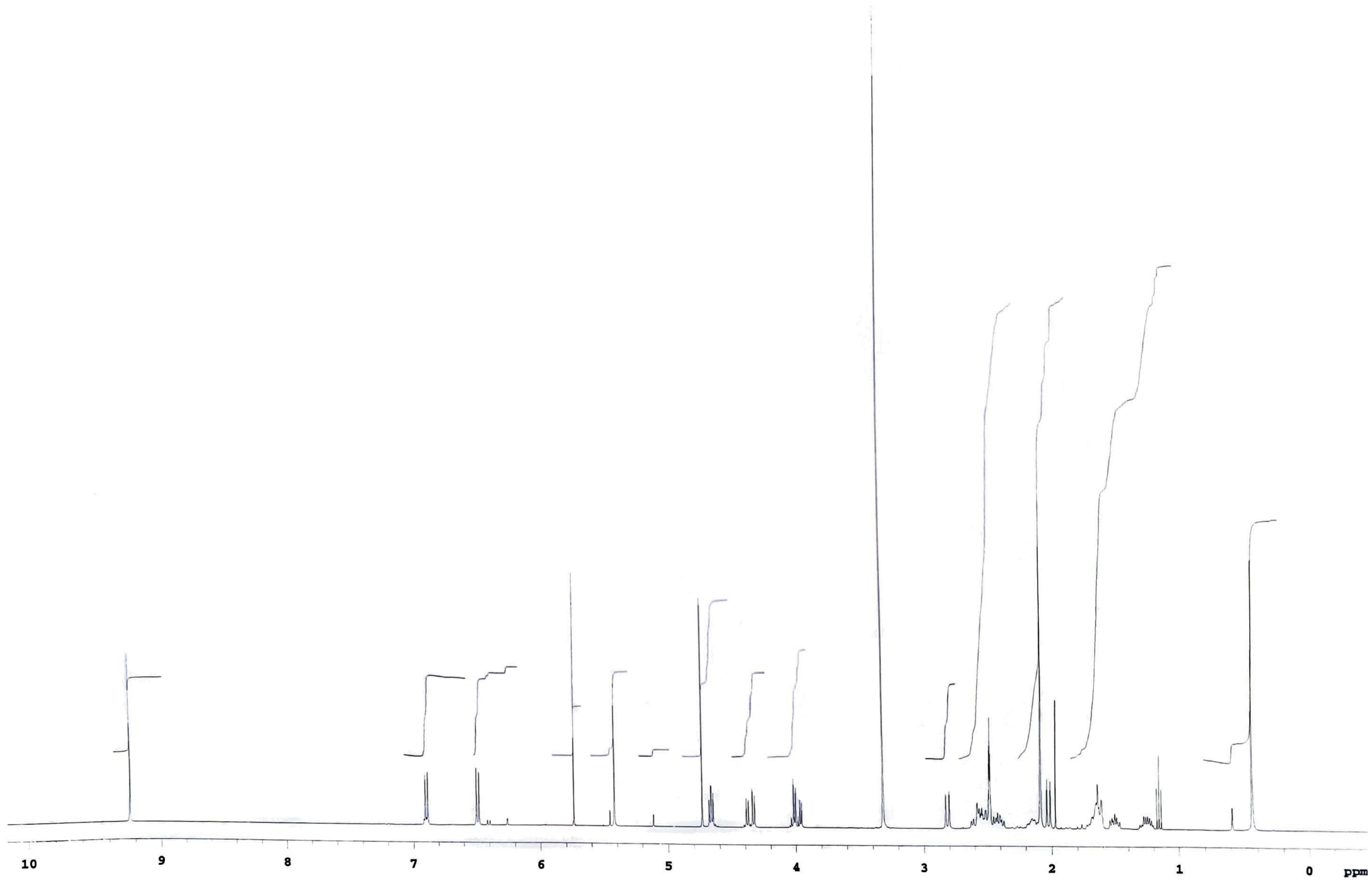

1H-NMR product 6

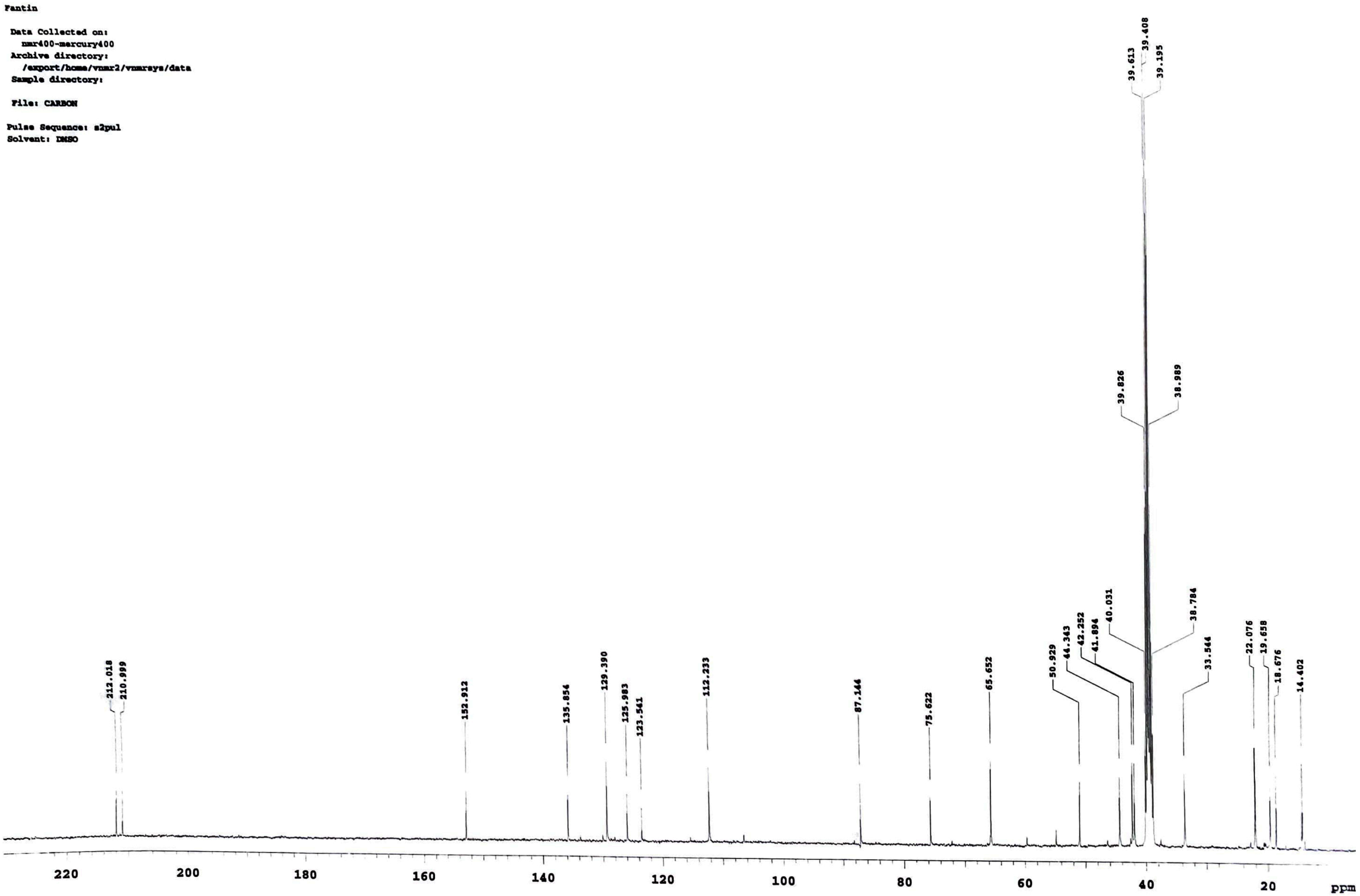

13C-NMR product 6

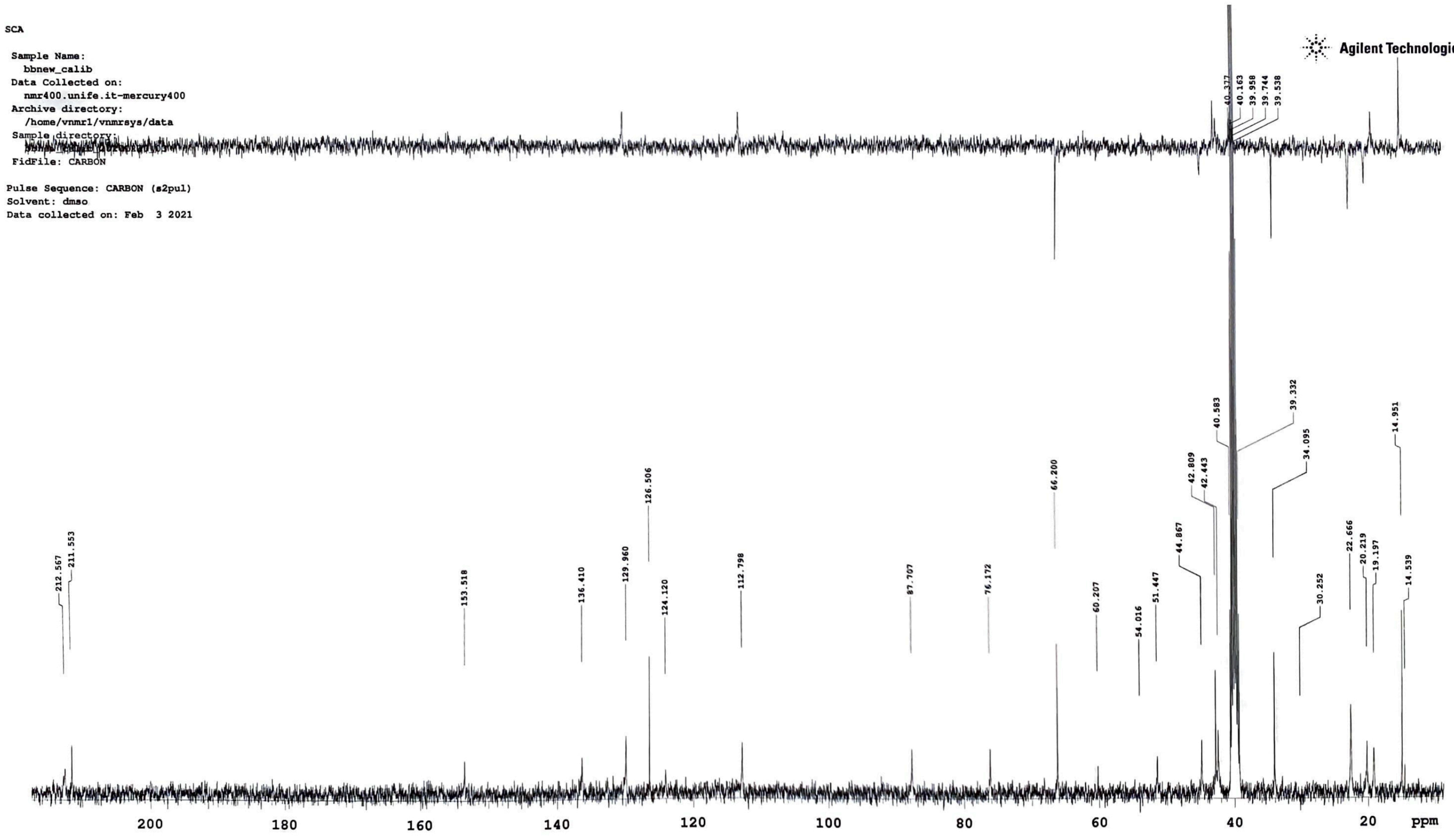

DEPT-NMR product 6

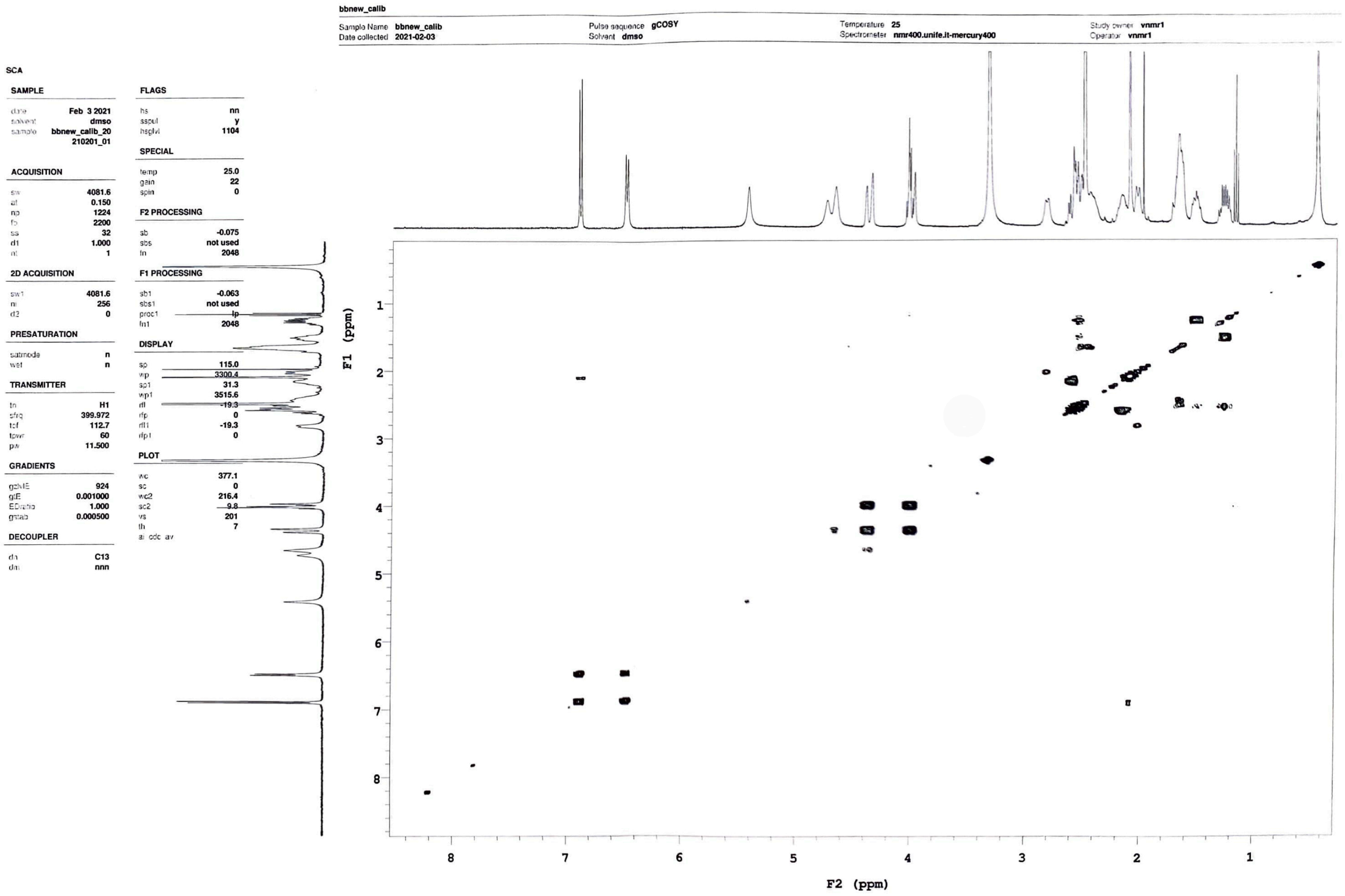

Data file exp

COSY-NMR product 6

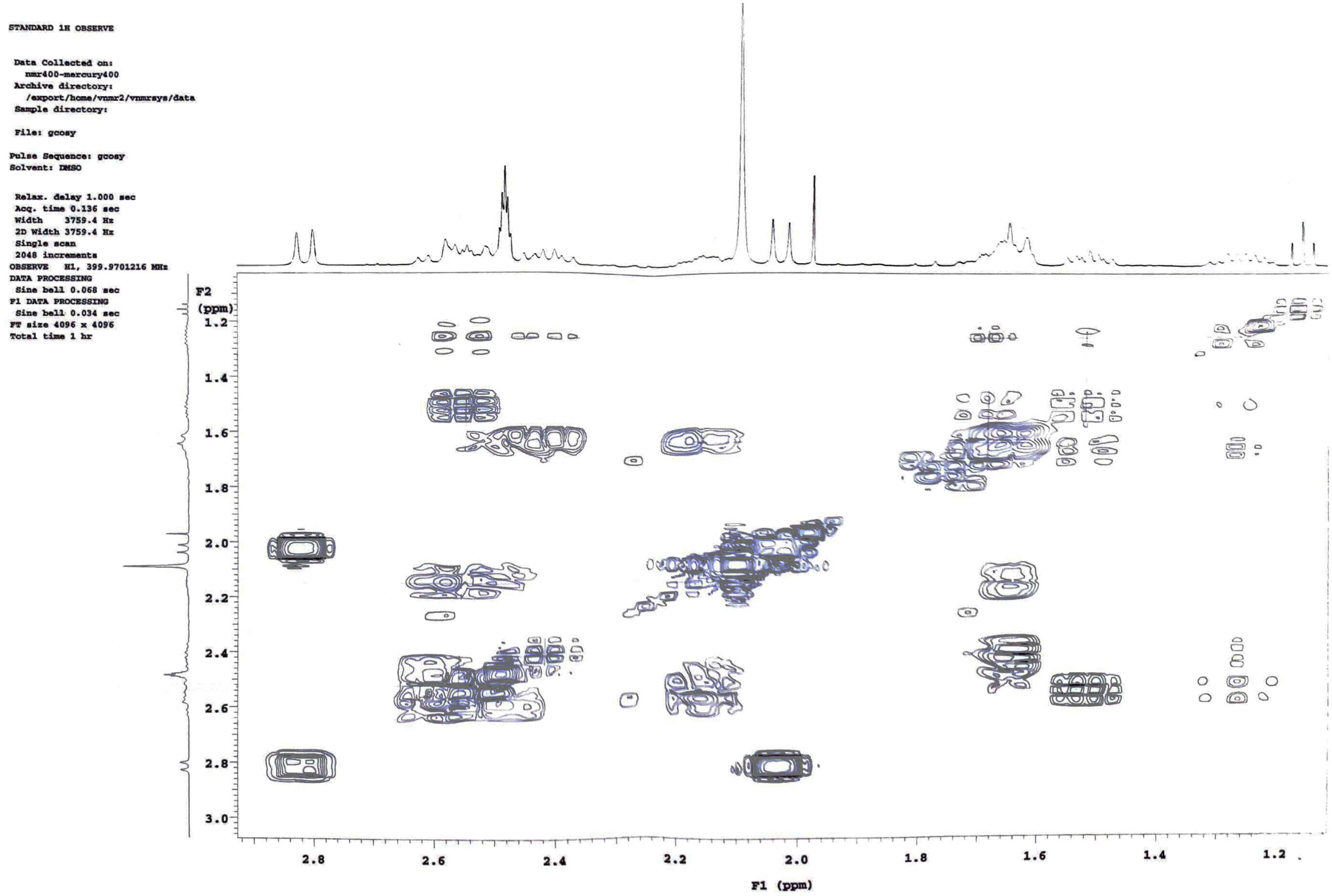

Focused COSY-NMR product 6

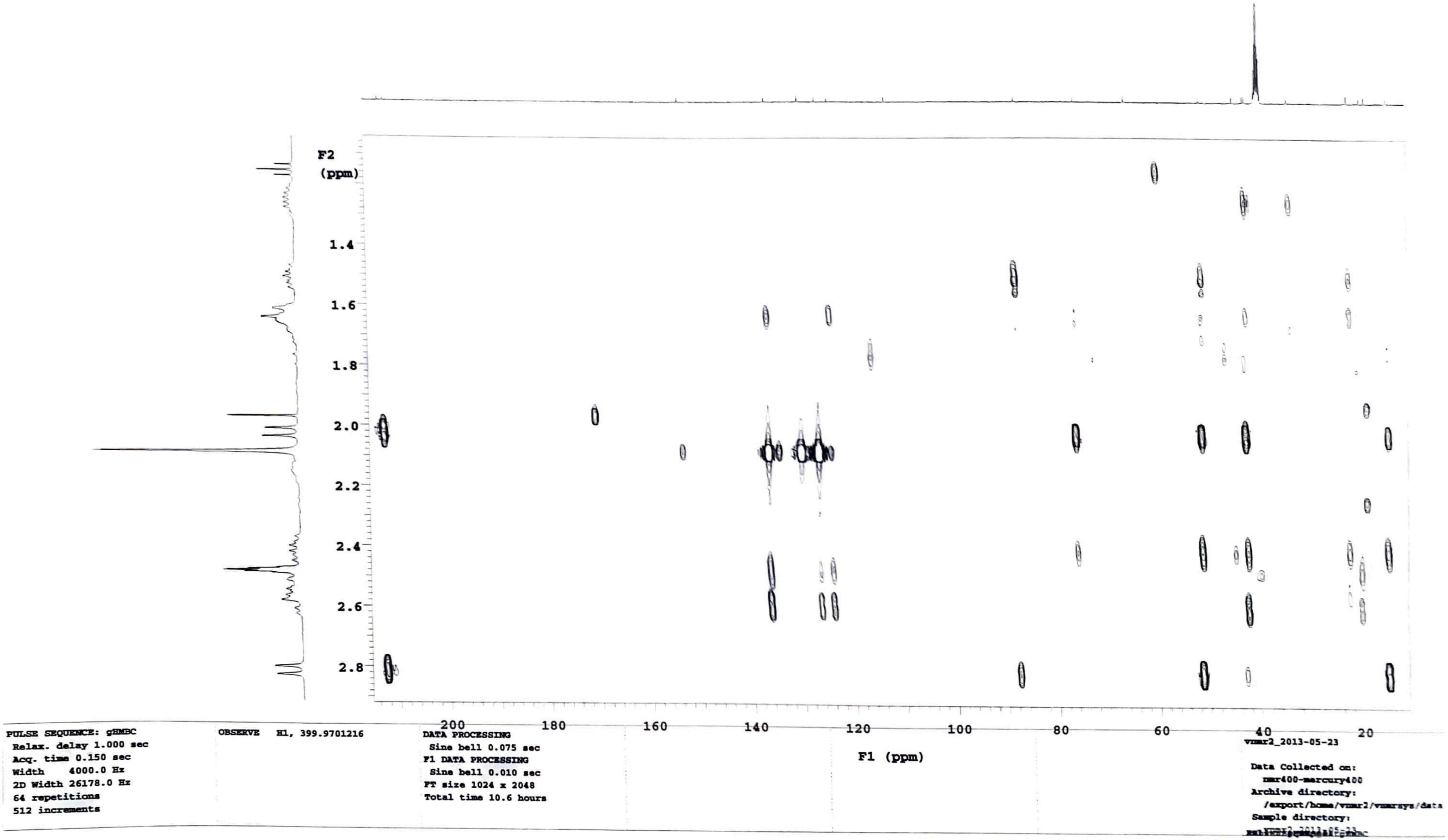

HMBC-NMR product 6

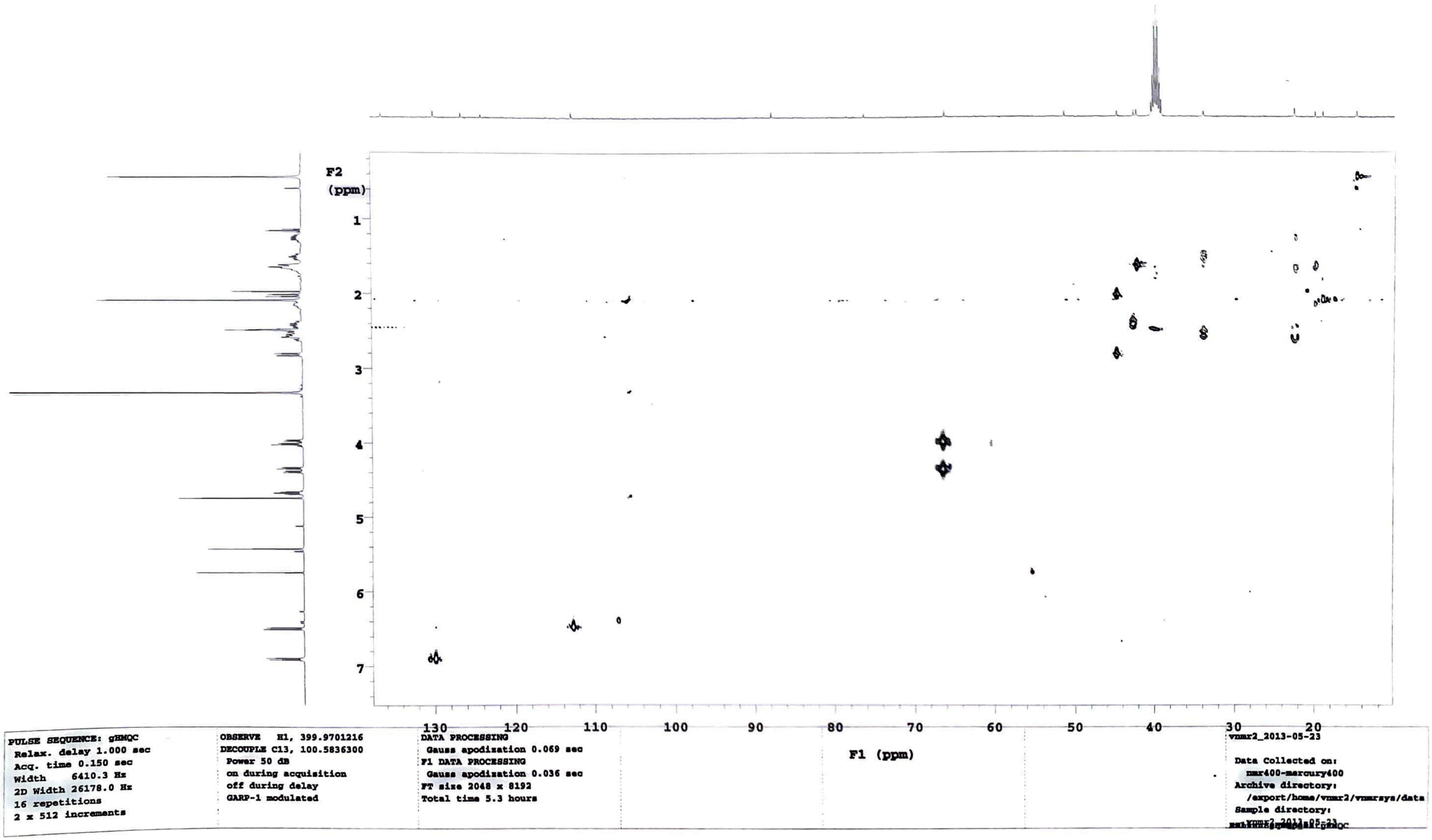

HMQC-NMR product 6

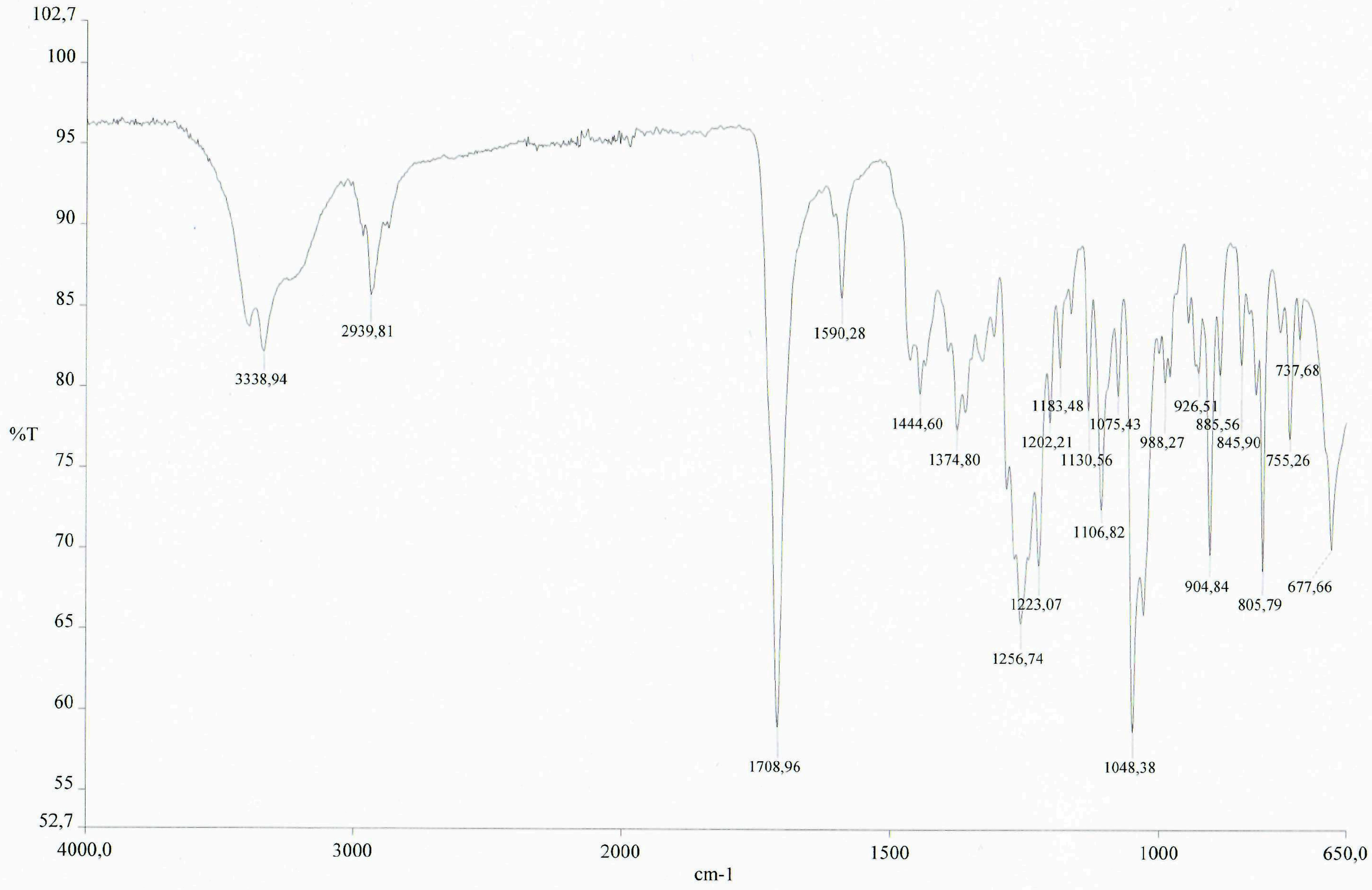
102,7
100
95
90
85
80
%T
75
70
65
60
55
52,7
4000,0
3000
2000
1500
1000
650,0
cm-1
3338,94
2939,81
1708,96
1590,28
1444,60
1374,80
1256,74
1223,07
1202,21
1183,48
1130,56
1106,82
1075,43
1048,38
988,27
926,51
904,84
885,56
845,90
805,79
755,26
737,68
677,66

IR product 6

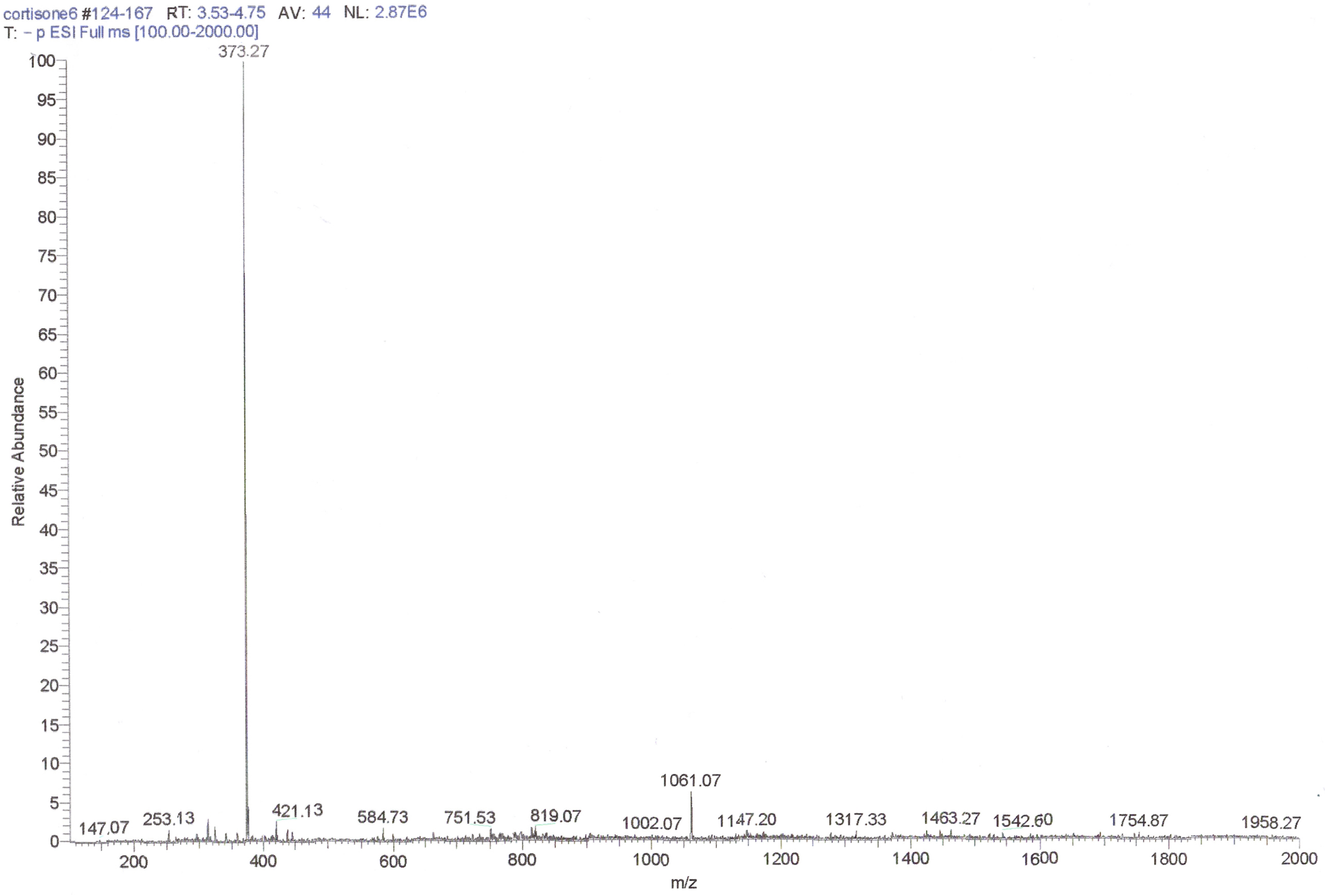
cortisone6 #124-167 RT: 3.53-4.75 AV: 44 NL: 2.87E6
T: - p ESI Full ms [100.00-2000.00]
373.27
1061.07
147.07
253.13
421.13
584.73
751.53
819.07
1002.07
1147.20
1317.33
1463.27
1542.60
1754.87
1958.27
Relative Abundance
m/z

MS product 6

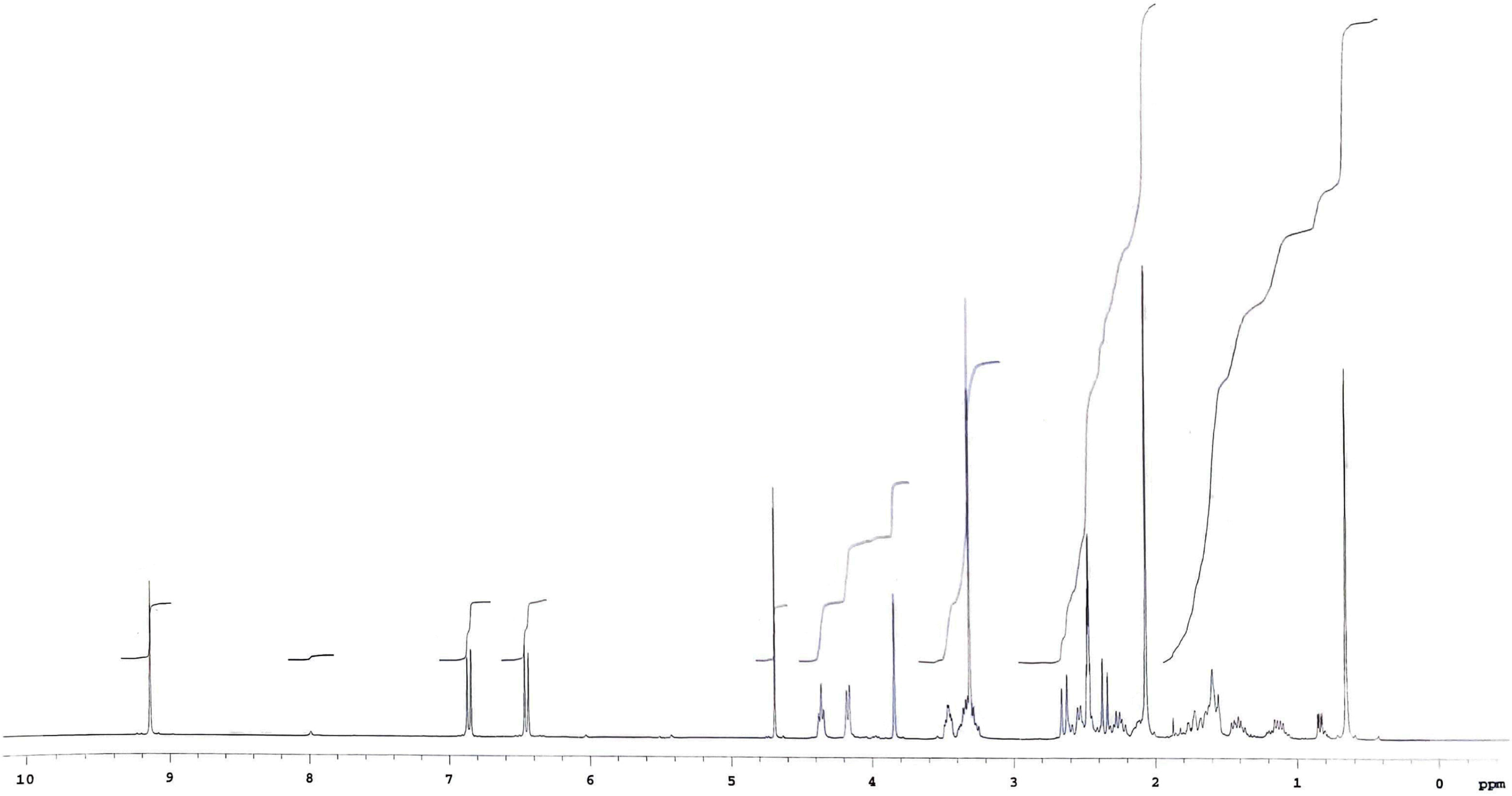

1H-NMR product 7

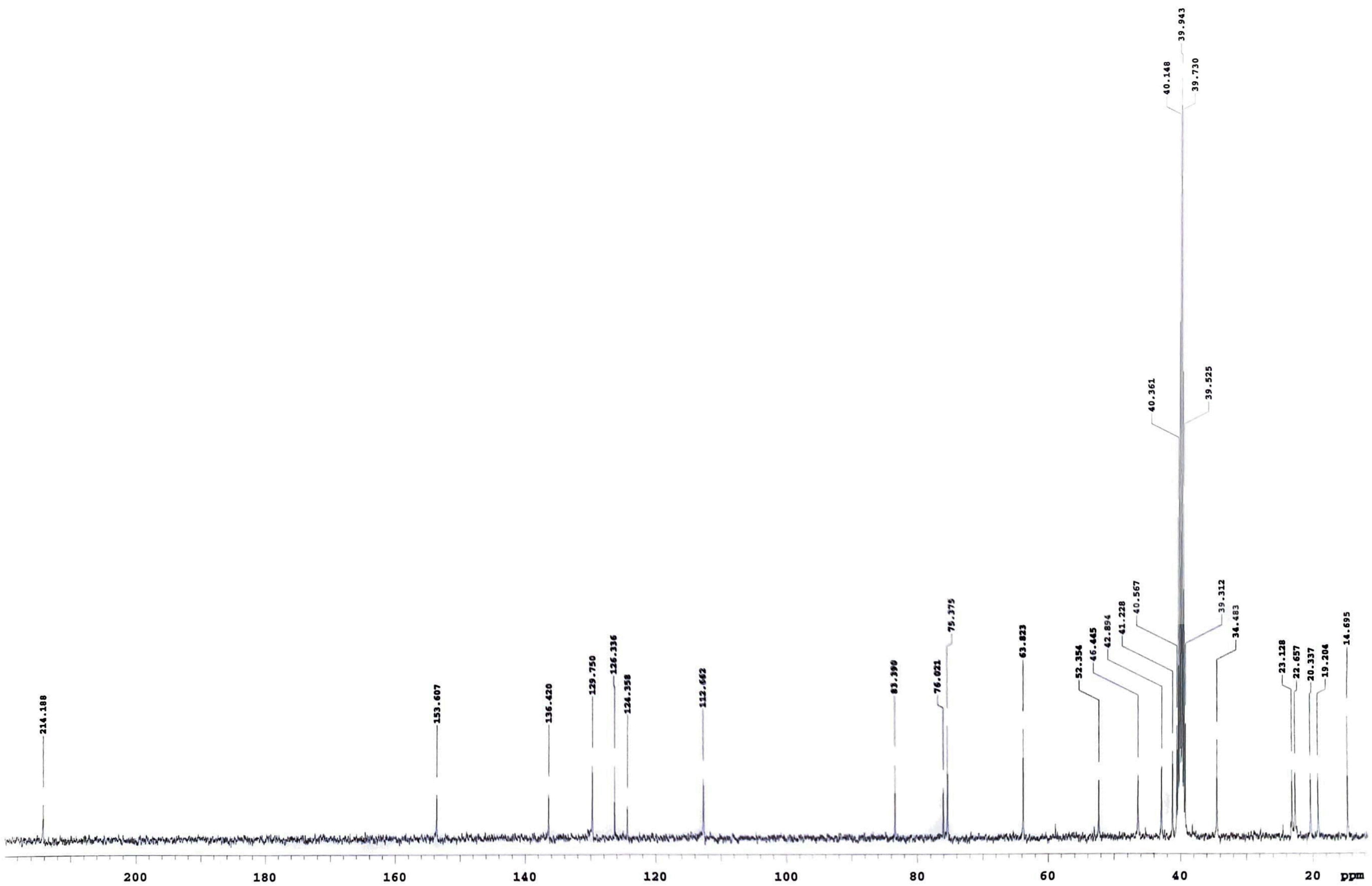

13C-NMR product 7

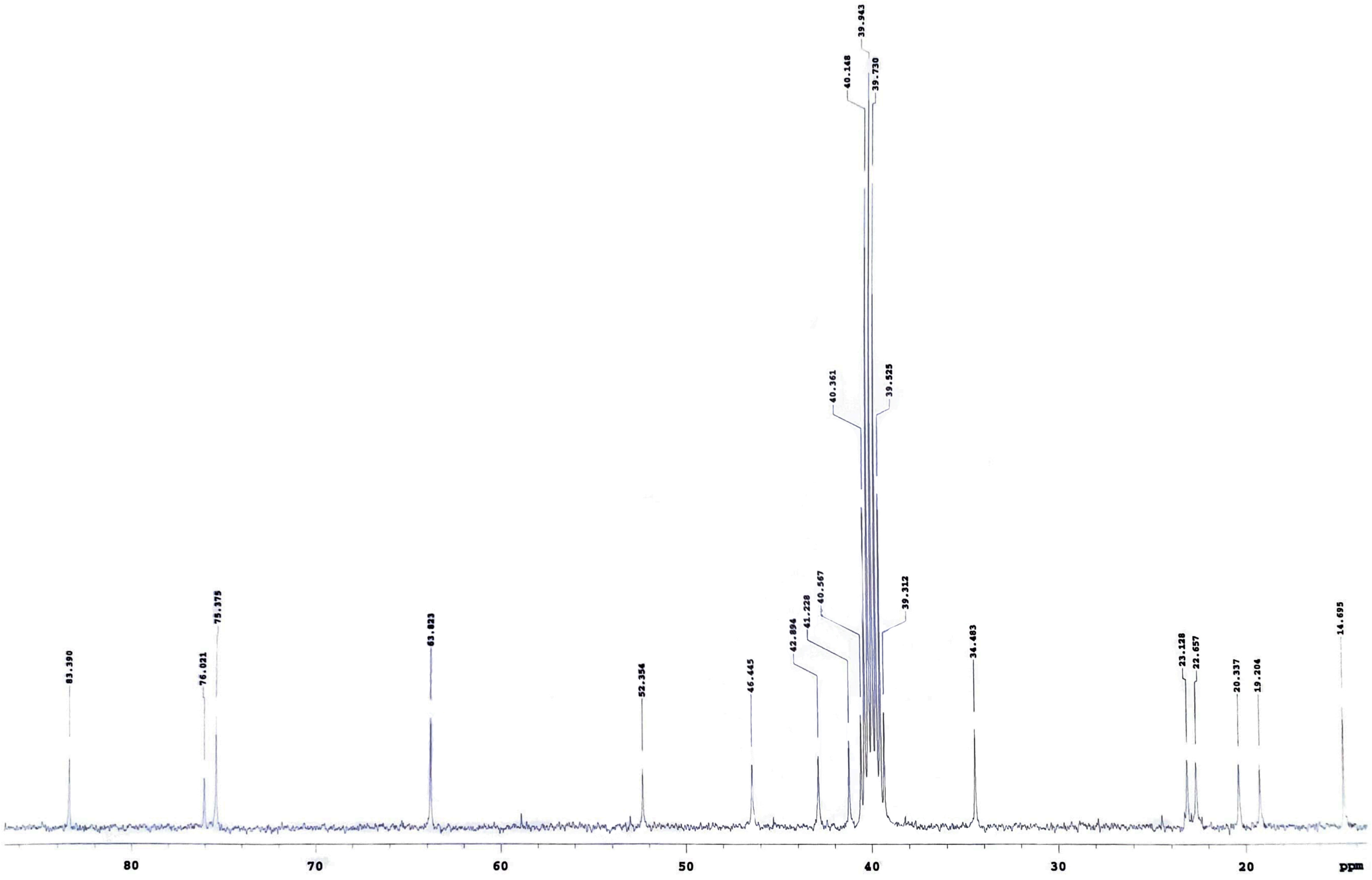

Focused 13C-NMR product 7

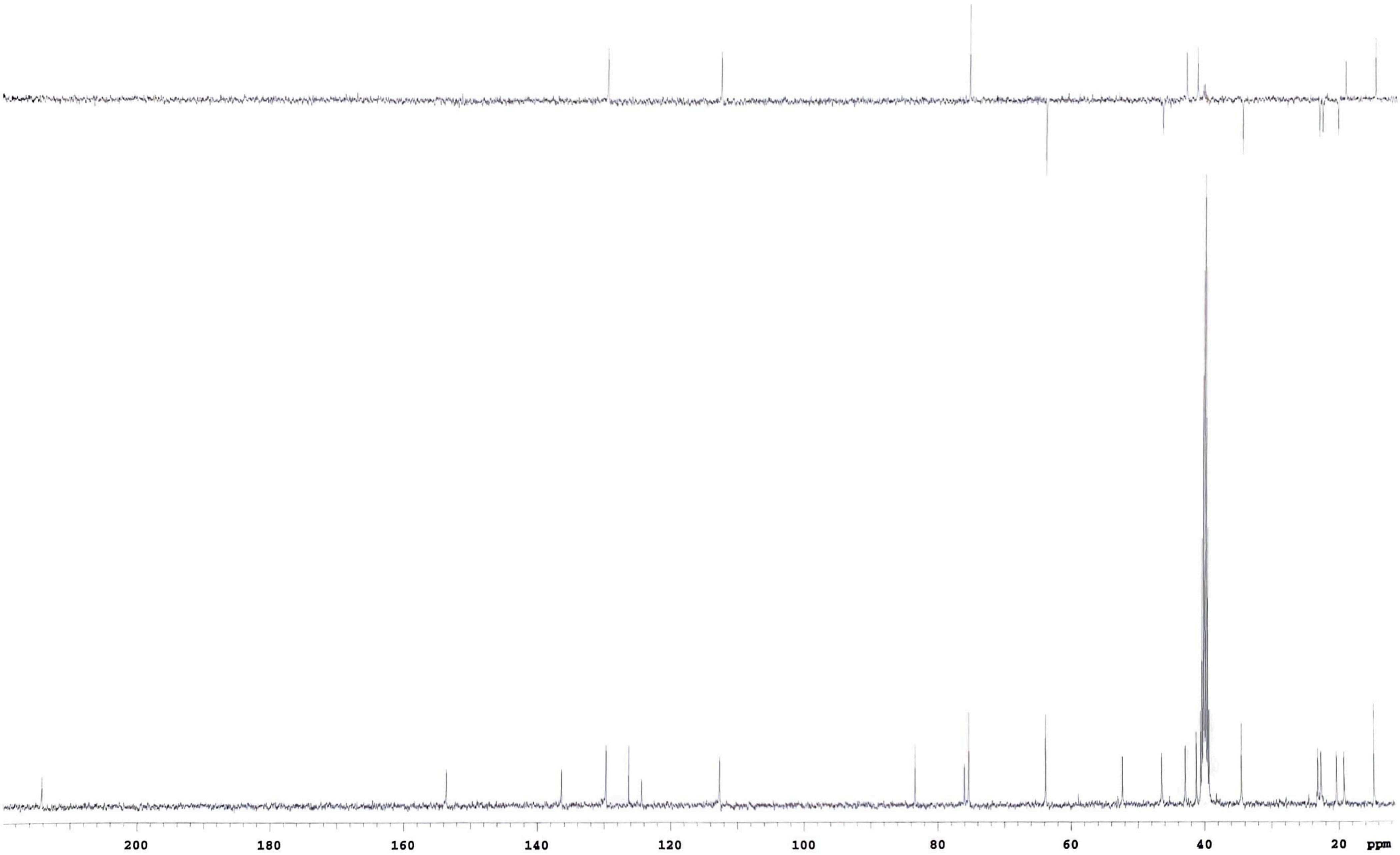

DEPT-NMR product 7

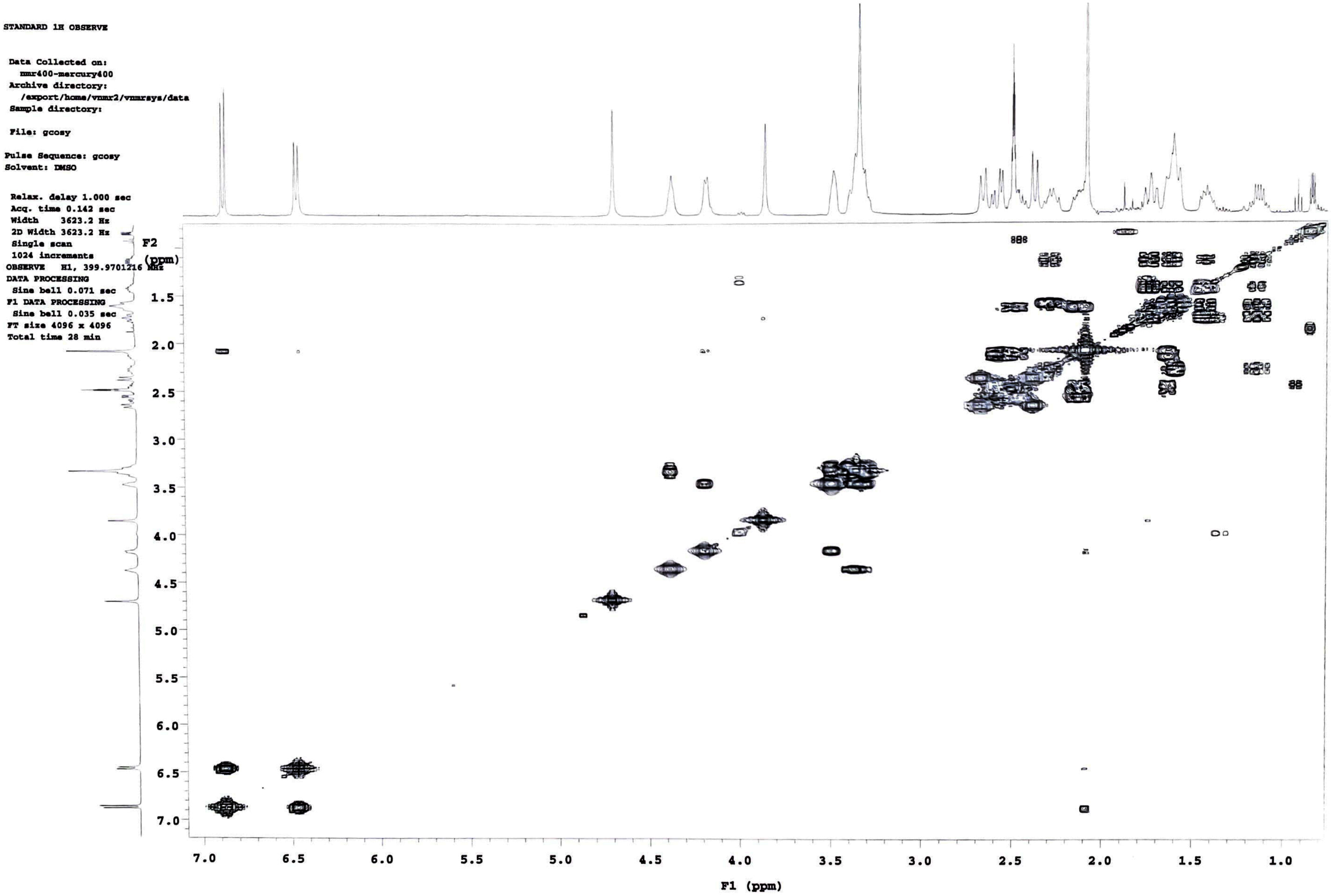

COSY-NMR product 7

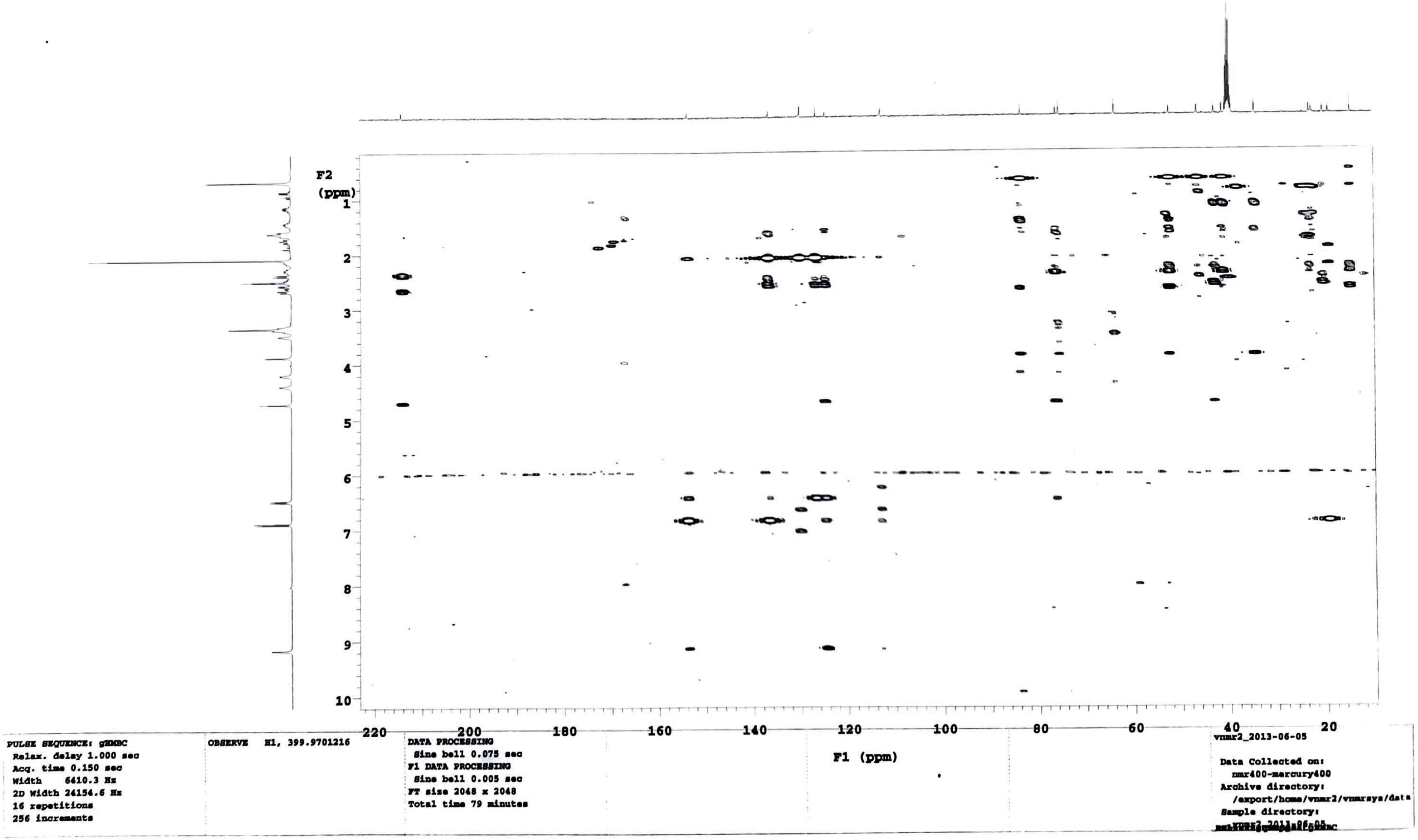

HMBC-NMR product 7

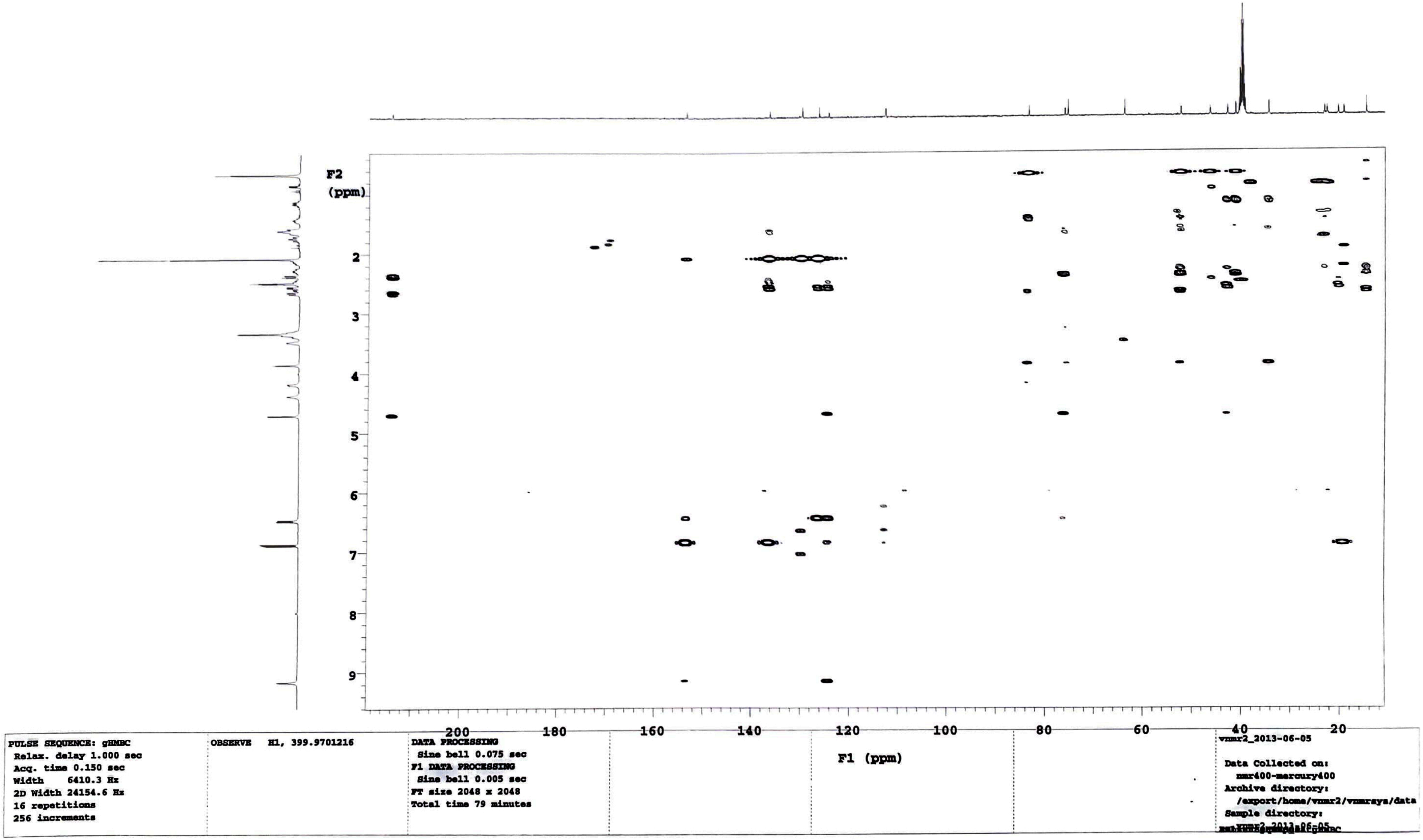

Focused HMBC-NMR product 7

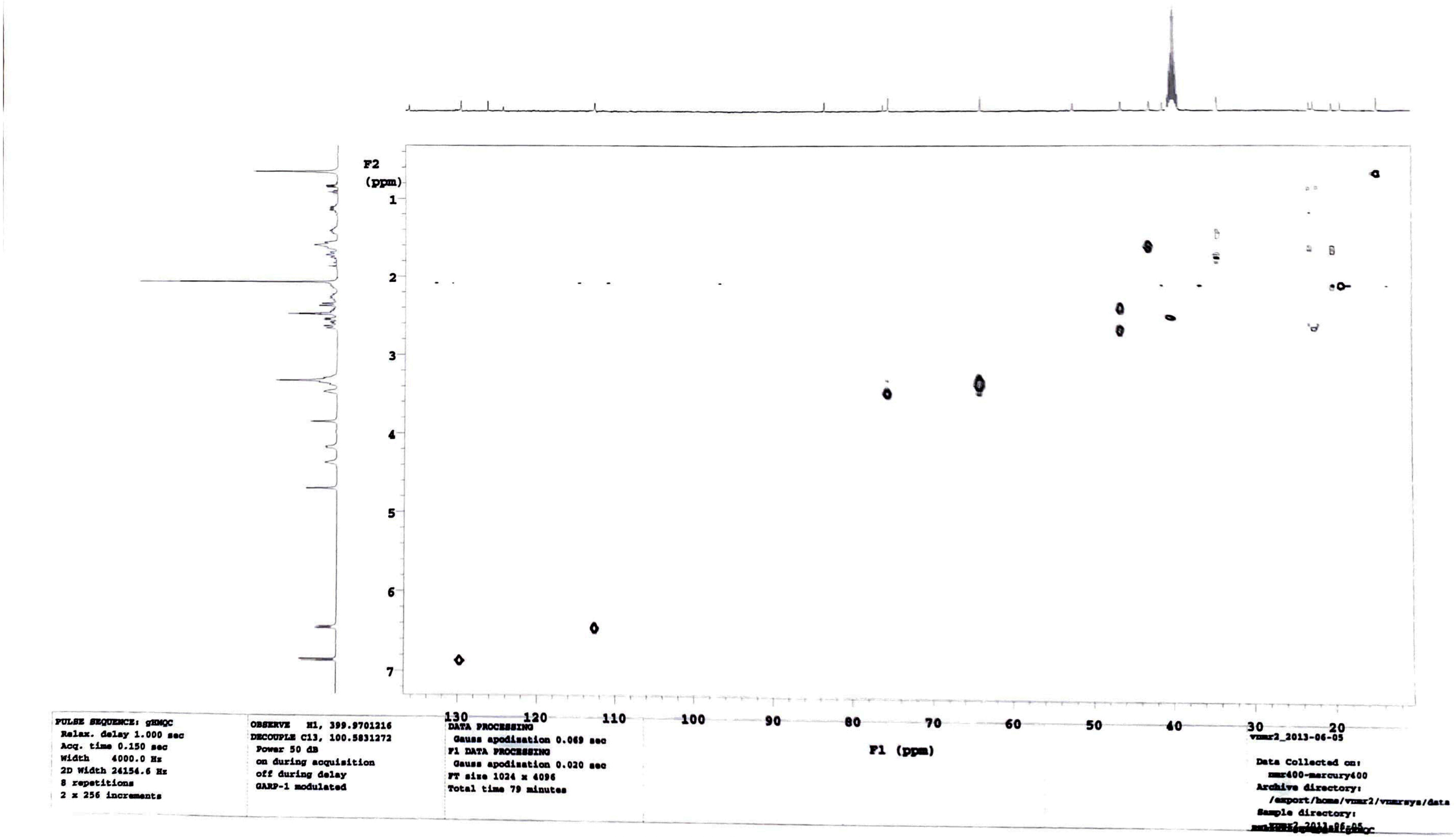

HMQC-NMR product 7

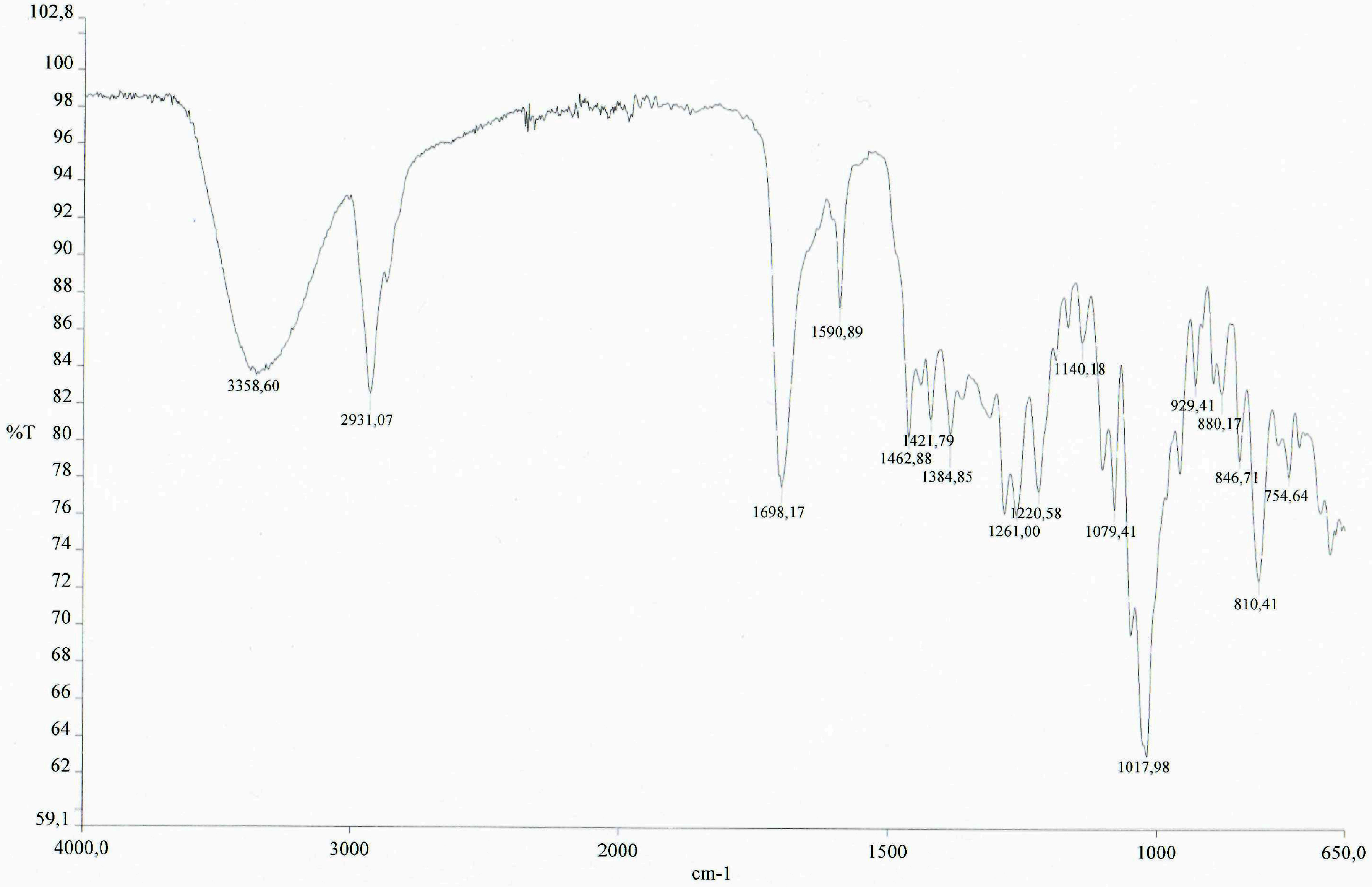
102,8
100
98
96
94
92
90
88
86
84
82
%T
80
78
76
74
72
70
68
66
64
62
59,1
4000,0
3000
2000
1500
1000
650,0
cm-1
3358,60
2931,07
1698,17
1590,89
1462,88
1421,79
1384,85
1261,00
1220,58
1140,18
1079,41
1017,98
929,41
880,17
846,71
810,41
754,64

IR product 7

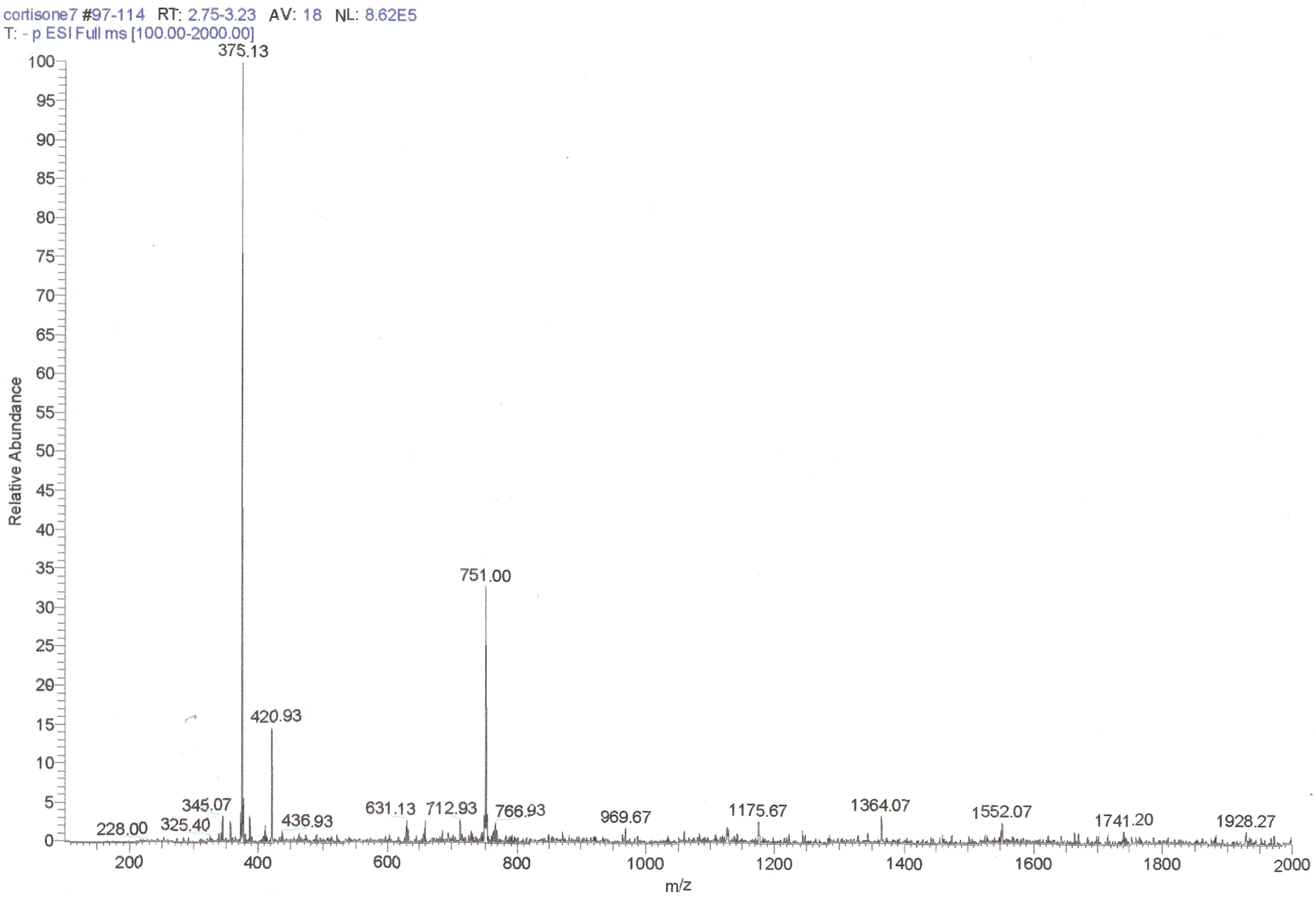
cortisone7 #97-114 RT: 2.75-3.23 AV: 18 NL: 8.62E5
T: - p ESI Full ms [100.00-2000.00]
375.13
751.00
420.93
345.07
325.40
228.00
436.93
631.13
712.93
766.93
969.67
1175.67
1364.07
1552.07
1741.20
1928.27
Relative Abundance
m/z

MS product 7
